# Comparative effectiveness of disease-modifying therapies for highly active relapsing-remitting multiple sclerosis despite previous treatment – a systematic review and network meta-analysis

**DOI:** 10.1186/s12883-025-04338-7

**Published:** 2025-08-09

**Authors:** Michael Köhler, Friedemann Paul, Kirsten Janke, Sibylle Sturtz, Daniela Preukschat, Sabine Ostlender, Michaela Florina Kerekes, Thomas Kaiser

**Affiliations:** 1 Institute for Quality and Efficiency in Health Care (IQWiG), Cologne, Germany; 2 Experimental and Clinical Research Center (ECRC), Charité– Universitätsmedizin Berlin, Berlin, Germany

**Keywords:** Multiple sclerosis, High disease activity, Data availability, Systematic review

## Abstract

**Background:**

Comparative assessments of all available disease-modifying therapies (DMTs) in patients with highly active relapsing-remitting multiple sclerosis (RRMS) are lacking, even though some of these DMTs are restricted to this MS subpopulation. We therefore aimed to compare DMTs in patients with highly active RRMS using re-analyses of individual patient data (IPD) provided by study sponsors.

**Methods:**

We searched for randomised controlled trials (RCTs) that included adult patients with RRMS and directly compared alemtuzumab, cladribine, dimethyl fumarate, fingolimod, natalizumab, ocrelizumab, ofatumumab, ozanimod, ponesimod and teriflunomide, or compared these DMTs with other drugs or placebo. Re-analyses of IPD for subpopulations of patients with high disease activity despite previous DMT were included in network meta-analyses (NMAs). As there is no widely accepted definition of high disease activity in RRMS, criteria were chosen to cover as wide a range of definitions as possible, while being sufficiently similar across studies.

**Results:**

We identified 14 relevant RCTs, including only 3 head-to-head comparisons of DMTs, and no relevant studies on natalizumab. All studies were pivotal studies for approval. The available re-analyses of IPD did not allow comprehensive NMAs. The main reasons for this were the overall paucity of RCTs, especially head-to-head comparisons, and a high risk of bias. In addition, data on patient-relevant outcomes and long-term follow-up (> 2 years) were lacking.

**Conclusion:**

Based on the largest possible evidence base, including previously unpublished data, our systematic review shows substantial evidence gaps for DMTs in highly active RRMS. This indicates a need for further research beyond regulatory requirements.

**Trial registration:**

Clinical trial number: not applicable.

**Supplementary Information:**

The online version contains supplementary material available at 10.1186/s12883-025-04338-7.

## Introduction

Multiple sclerosis (MS) is one of the most common neurodegenerative diseases, and often has a lifelong, debilitating impact on patients. The most common form, relapsing-remitting multiple sclerosis (RRMS), accounts for approximately 85% of cases [1, 2]. It is characterised by repeated episodes of MS-specific symptoms (relapses), including but not limited to fatigue and motor, visual and cognitive impairment. Relapses are often associated with disability progression (e.g. loss of motor function) [1]. A subgroup of RRMS patients have a more aggressive disease course known as highly active MS, characterised by rapid physical and cognitive deterioration despite previous disease-modifying therapy (DMT), i.e., treatment with one or more disease-modifying drugs that target the underlying cause of the disease [3].

The first generation of DMTs for RRMS was introduced in the 1990s, with beta-interferons and glatiramer acetate still being important treatment options [4]. Since 2006, a number of DMTs for RRMS with novel mechanisms of action have become available in the European Union (EU), including alemtuzumab, cladribine, dimethyl fumarate, fingolimod, natalizumab, ocrelizumab, ofatumumab, ozanimod, ponesimod and teriflunomide [5–14]. Some – cladribine, fingolimod, alemtuzumab and natalizumab – are restricted to patients with highly active RRMS [7, 8, 11]. All 4 drugs have been associated with serious, sometimes fatal, adverse effects which lead to restrictions of the European market authorisation. Severe thyroid effects, immune thrombocytopenic purpura, nephropathies, cardiovascular disease and other adverse effects, including fatal cases, have been reported with alemtuzumab [15]. Some of these cases may have occurred late after administration. Long-term follow-up of patients for 48 months after the last infusion is therefore required [16]. In addition, cases of progressive multifocal leukoencephalopathy (PML) have been reported with natalizumab [17]. Both drugs have therefore been restricted to patients with highly active or rapidly progressing disease despite previous DMT [16, 17]. For cladribine and fingolimod serious, sometimes fatal adverse effects have also been observed, and the label of these drugs initially included patients with highly active disease only [18, 19]. However, to date there have been no systematic comparisons of the benefits and harms of DMTs in this MS subpopulation. Previous systematic reviews have mostly included patients with RRMS regardless of disease activity or have not considered all of the drugs mentioned above [20–25]. The Federal Joint Committee (G-BA), the highest decision-making body in the German health care system, therefore commissioned the Institute for Quality and Efficiency in Health Care (IQWiG), the German health technology assessment (HTA) agency, to conduct an HTA with a systematic review of the comparative effectiveness of alemtuzumab, cladribine, dimethyl fumarate, fingolimod, natalizumab, ocrelizumab, ofatumumab, ozanimod, ponesimod and teriflunomide in the target population of patients with highly active RRMS despite previous DMT.

In the present article we report the results of this review. The full report (in German) and protocol are available on the IQWiG website [26, 27], including an English translation of the core report [28].

## Materials and methods

IQWiG’s general HTA methods are described in its methods paper [29]. We also followed the PRISMA NMA extension for reporting systematic reviews with NMAs [30].

### Inclusion criterion: high disease activity

There is no generally accepted definition of highly active RRMS. Current MS guidelines [31–37] and reviews [3, 38, 39] either do not provide a clear definition or list several competing definitions, based either on different operationalisations of relapse frequency, occurrence of brain lesions, disability progression or disability severity at diagnosis, or combinations of these features. Similarly, the Summaries of Product Characteristics do not provide a consistent definition. Instead, they use definitions of disease activity pre-specified in the pivotal studies of each DMT. High disease activity is usually defined by the relapse rate and new CNS lesions on magnetic resonance imaging (MRI). Definitions in clinical studies vary, but are usually based on the occurrence of at least one relapse in the previous year, combined with the occurrence of multiple T2-weighted lesions and/or at least one gadolinium-enhancing (Gd+) T1-weighted lesion, despite previous DMT.

In this systematic review, we proposed an approximate definition of high disease activity based on patient-relevant features and MRI characteristics. Our definition was based on 3 manifestations of disease activity:

1. a purely clinical manifestation independent of MRI activity, defined as ≥ 1 relapse with severe functional impairment in the past 12 months or ≥ 2 such relapses in the past 24 months;
2. a mixed clinical and MRI manifestation consisting of ≥ 1 relapse in the past 12 months or ≥ 2 relapses in the past 24 months, each associated with ≥ 3 new or enlarged T2-lesions or ≥ 1 Gd+ T1 lesion;
3. an MRI-only manifestation, requiring the appearance of numerous new or enlarged lesions in the past 12 months, but no documentation of relapse activity.

We included the third definition because we assumed that many new or enlarged lesions were indicative of a possibly undocumented clinical manifestation. As T2-lesions persist over time, only new or enlarged T2-lesions were included in the assessment of current activity. Gd+ T1 lesions do not persist for more than 4 to 6 weeks, so any T1 activity indicates current inflammatory activity. For all manifestations included in our definition, a sufficiently highly dosed and complete course of treatment with at least one DMT was required before high disease activity could be diagnosed. This previous treatment had to take into account the latency period of the DMT (e.g. 3 months for interferons), in order for the treatment to be fully effective. In addition, no more than 12 months should have elapsed between the end of the last DMT and the start of study treatment.

### Selection of studies

We searched for randomised controlled studies (RCTs) that included adult patients with RRMS who met any of the 3 criteria for high disease activity described above. Regardless of the definition of disease activity, all patients had to have received at least one adequate initial course of DMT. This means that a diagnosis of high disease activity could only be made when the previous treatment was fully effective, i.e. after at least 3 to 6 months of treatment. Possible initial treatments included interferon-β 1a, interferon-β 1b, glatiramer acetate, dimethyl fumarate and teriflunomide, administered according to the European Medicines Agency (EMA) label.

Patients could be treated with any of the 10 DMTs according to the EMA label. As they were to be compared with each other, we only included studies that compared one of these DMTs directly or with another drug or placebo that could serve as a common comparator in a network meta-analysis (NMA), i.e. that was used as a comparator for at least 2 DMTs of interest. Because disability progression in MS occurs over a longer period of time, the minimum follow-up period had to be at least 2 years for at least a part of the study population.

We analysed the comparative effectiveness using patient-relevant outcomes. Therefore, we excluded MRI-based assessments of disease activity alone and restricted our analyses to the following outcomes: overall survival; relapses (annual relapse rate [ARR], number of patients with ≥ 1 confirmed relapse); disability progression according to the Expanded Disability Status Scale (EDSS), to be confirmed after 24 weeks; disability severity according to the Multiple Sclerosis Functional Composite (MSFC) status; walking ability; fatigue; visual impairment; and health-related quality of life (HRQoL). Harms were assessed by means of serious adverse events (SAEs), treatment discontinuation due to adverse events (AEs) and specific AEs (PML, serious infections, serious neoplasms and serious secondary autoimmune disorders).

As the main source of study data (including unpublished data), we asked the marketing authorisation holders (MAHs) of the 10 DMTs to provide us with complete information on all studies of the drugs analysed, as well as complete clinical study reports (CSRs) for the studies (see Supplementary Text 1 for further details). In addition, we searched clinical study registries (ClinicalTrials.gov, EU Clinical Trials Register, ICTRP), Medline and the G-BA database of early benefit assessments in Germany to identify other relevant studies that may not have been identified in the documents provided by the MAHs. Only documents in English or German were included. Two reviewers independently screened the titles and abstracts of the retrieved citations to identify potentially eligible publications. They also independently assessed the full texts. All documents retrieved from non-bibliographic sources were also screened by 2 reviewers for eligibility or relevant study information. Disagreements were resolved by consensus.

If a study population was not restricted to our target population, we asked the study sponsors to provide re-analyses of individual patient data (IPD) to allow separate results for the target population.

### Data extraction

Data extraction was performed by 2 reviewers; one extracted the data and the other checked the extracted data. Disagreements were resolved by consensus. The main sources for extraction were the study data provided by the sponsors. Where necessary, we used the requested subpopulation data.

For each relevant study, we extracted the following information: study characteristics (citation, study design and duration, sample size, location, number of centres, study period, primary and relevant secondary outcomes); intervention characteristics; inclusion and exclusion criteria according to the study protocol; baseline characteristics of patients in the relevant subpopulation (demographic and disease-specific characteristics, including disease activity at baseline and prior and concomitant drug treatment); results for patient-relevant outcomes; and relevant items for assessing risk of bias.

The full datasets extracted from each study are available in the full German-language report [27].

### Assessment of risk of bias and certainty of conclusions

Two reviewers assessed the risk of bias for all relevant studies using criteria commonly applied to RCTs [40–49]. According to IQWiG’s methods [29], if an indirect comparison is based on only one study per intervention, a statistically significant effect for an indirect comparison is only relevant if the single study has a low risk of bias. If more than one study with a high risk of bias contributes to the contrast, the effect is considered relevant regardless of the risk of bias. At the level of individual outcomes, the risk of bias was only assessed if it was possible to compare at least 2 different DMTs. In this case, the risk of bias was assessed for the studies with results for all outcomes that could be compared.

The certainty of the conclusions of an NMA was determined by the number of studies that informed the pairwise comparisons, the inclusion of direct (i.e., head-to-head) comparisons, the homogeneity of the studies, the consistency of the direct and indirect comparisons, and the risk of bias of the studies contributing to an effect.

### Data synthesis and statistical methods

We aimed to compare several DMTs simultaneously using an NMA. In cases where an NMA was not possible, pairwise direct comparisons between DMTs within the included studies were to be used. When neither an NMA nor direct comparisons were possible, pairwise adjusted indirect comparisons were to be performed according to Bucher [50]. Statistical analyses were based on intention-to-treat analyses as described in the study reports. The prerequisite for conducting an NMA was adequate structural quality of the study pool, i.e. a study pool that met the assumptions of similarity, homogeneity and consistency. The requirements for these 3 criteria are described in Supplementary Text 2.

The following characteristics of the studies or study populations were considered to check the assumptions of similarity: age, sex, region (Organisation for Economic Co-operation and Development [OECD] versus non-OECD countries), previous DMT, disease severity, disease duration of the target population, intervention, concomitant medication, study duration, study year, and the outcomes considered. Homogeneity was assumed if there was no substantial heterogeneity in the study pool for a given contrast that included 2 or more studies. Consistency was assumed if the estimates from an indirect comparison were confirmed by the estimates from a direct comparison in a closed loop of the network. If any of the assumptions were rejected, no NMA was performed. If homogeneity or consistency could not be assessed because of the structure of the network or the number of studies, the NMA was still performed, but the certainty of the conclusion was downgraded. Heterogeneity was assessed by an interaction test. Where possible, inconsistency was tested locally within each loop using the node-splitting procedure [51].

Details on effect measures, relevance of effects and planned analyses are provided in Supplementary Text 2.

### Patient and public involvement

Patients and the general public were involved in the full HTA according to IQWiG’s methods [29]. Before publishing our protocol, we invited patients with MS to a meeting to discuss their experiences of the disease. Seven participants described the symptoms that were important to them, their experiences of different treatments and their side effects as well as their personal treatment goals and preferences. After the publication of both the preliminary protocol and preliminary report, a public commenting procedure was held and the comments received were taken into account in the revised final versions [26, 27]. The submitted statements are available online [52, 53]. The changes made to the preliminary versions are described in Supplementary Text 3.

## Results

### Relevant studies and subpopulations

**Fig. 1:**
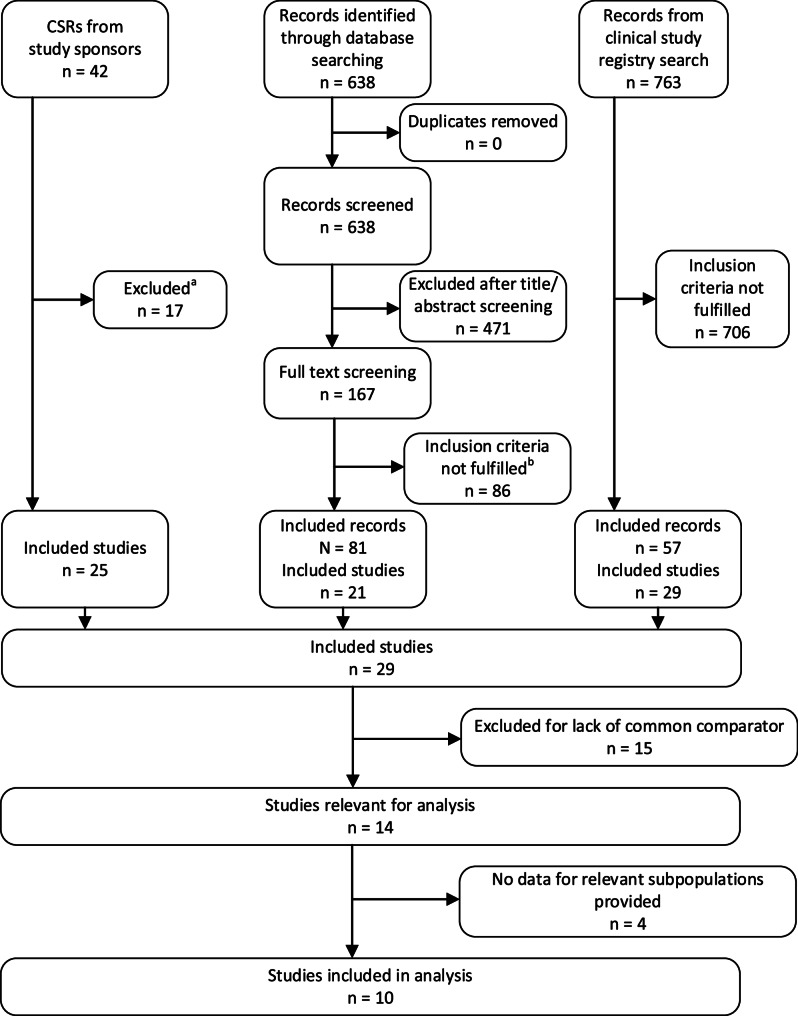
Flowchart of information retrieval **a**: Reasons for exclusion were wrong population (*n* = 3), wrong intervention (*n* = 3), study duration less than 2 years (*n* = 11); **b**: Reasons for exclusion were wrong population (*n* = 23), wrong intervention (*n* = 6), wrong comparator (*n* = 1), no RCT (*n* = 5), study duration less than 2 years (*n* = 12), no full publication available, only conference abstracts, posters etc. (*n* = 39); CSR = clinical study report

29 studies met the inclusion criteria for our review (Fig. 1). Most of these studies were identified by screening the documents provided by study sponsors. Four studies were identified by searching study registries, as they did not investigate any of the 10 DMTs listed in the protocol as an intervention, but as a comparator. Of the 29 studies, 14 were included in direct or indirect comparisons of different DMTs, because they had a common comparator with at least one other DMT or provided a direct comparison of 2 DMTs. As shown in Table 1, the 14 relevant studies investigated alemtuzumab (*n* = 1), cladribine (*n* = 1), dimethyl fumarate (*n* = 2), fingolimod (*n* = 2), ocrelizumab (*n* = 2), ofatumumab (*n* = 2), ozanimod (*n* = 1), ponesimod (*n* = 1) and teriflunomide (2 studies comparing teriflunomide with placebo and 3 studies comparing teriflunomide with either ofatumumab or ponesimod). None of the studies of natalizumab, including the pivotal AFFIRM and SENTINEL studies [54, 55] and the REVEAL study [56], met our inclusion criteria. The AFFIRM study included only treatment-naïve patients, while in the SENTINEL study natalizumab was administered in combination with interferon, which is not an approved regimen according to the EMA label for natalizumab [11]. The REVEAL study did not meet the inclusion criterion of a minimum follow-up period of 2 years. One additional study of natalizumab met our inclusion criteria, but was not included in the comparative NMAs due to a lack of common comparators with other DMTs. All relevant studies were pivotal studies conducted for the approval of the DMTs. We did not identify any post-approval studies.

**Table 1:** Size of study populations and target populations included in analyses for comparison of DMTs in patients with highly active RRMS despite previous DMT

Comparison	Population size					
Study^a^	Study population		Pre-treated with DMT		Highly active disease despite previous DMT^b^	
	*N*_Intervention_	*N*_Comprator_	*N*_Intervention_ (%)	*N*_Comparator_ (%)	*N*_Intervention_ (%)	*N*_Comparator_ (%)
**DMT vs. IFN-β 1a**						
Alemtuzumab vs. IFN-β 1a						
CARE-MS II	436	231	426 (98)	202 (87)	363 (83)	199 (86)
Ocrelizumab vs. IFN-β 1a						
OPERA I	410	411	107 (26)	117 (29)	n.r.	n.r.
OPERA II	417	418	113 (27)	103 (25)	n.r.	n.r.
Ozanimod vs. IFN-β 1a						
RADIANCE B	433	441	123 (28)	126 (27)	17 (4)	17 (4)
**DMT vs. placebo**						
Cladribine vs. placebo						
CLARITY	433	437	113 (26)	142 (32)	13 (3)	17 (4)
Dimethyl fumarate vs. placebo						
CONFIRM	359	363	101 (28)	111 (31)	n.r.	n.r.
DEFINE	410	408	162 (40)	172 (42)	n.r.	n.r.
Fingolimod vs. placebo						
FREEDOMS	425	418	181 (43)	169 (40)	34 (8)	28 (7)
FREEDOMS II	358	355	264 (74)	259 (73)	75 (21)	69 (19)
Teriflunomide vs. placebo						
TEMSO	359	363	102 (28)	90 (25)	39 (11)	32 (9)
TOWER	372	389	126 (34)	135 (35)	66 (18)	68 (17)
**DMT vs. DMT**						
Ofatumumab vs. teriflunomide						
ASCLEPIOS I	465	462	274 (59)	280 (61)	121 (26)	122 (26)
ASCLEPIOS II	481	474	286 (59)	293 (62)	135 (28)	147 (31)
Ponesimod vs. teriflunomide						
OPTIMUM	567	566	242 (43)	245 (43)	33 (6)	45 (8)
Natalizumab	No relevant studies were identified.					

*DMT* disease modifying therapy, *IFN *interferon, *N *number of patients per study/population, *n.r*. not reported, *RRMS *relapsing-remitting multiple sclerosis

^a^For references of the available studies, see Supplementary Table 1

^b^For information on the definition on highly active disease despite previous DMT in the individual studies, see Supplementary Table 2

All studies included patients with RRMS or relapsing MS (RMS), but with a wider range of disease activity and previous treatments than specified in our review. For our target population, the MAHs provided re-analyses of IPD for all DMTs except for dimethyl fumarate and ocrelizumab. Therefore, the following 4 studies could not be included in our analysis: CONFIRM, DEFINE (relevant comparison in both studies: dimethyl fumarate versus placebo), OPERA I and II (ocrelizumab versus IFN-β 1a), leaving subpopulations of 10 studies and 7 DMTs for comparison (see Supplementary Table 2 for the definitions of the relevant subpopulations as applied by the study sponsors). As shown in Table 1, only a small part of the study populations matched the definition of the target population. In most studies, only 30–75% of the total study population had previously been treated with a DMT. Together with the criterion of high disease activity, this resulted in very small target populations in some cases, e.g. less than 10% of the total study population in the studies CLARITY, FREEDOMS, OPTIMUM and RADIANCE B. In the end, our review included 1640 patients in the target population.

### Network characteristics

**Fig. 2:**
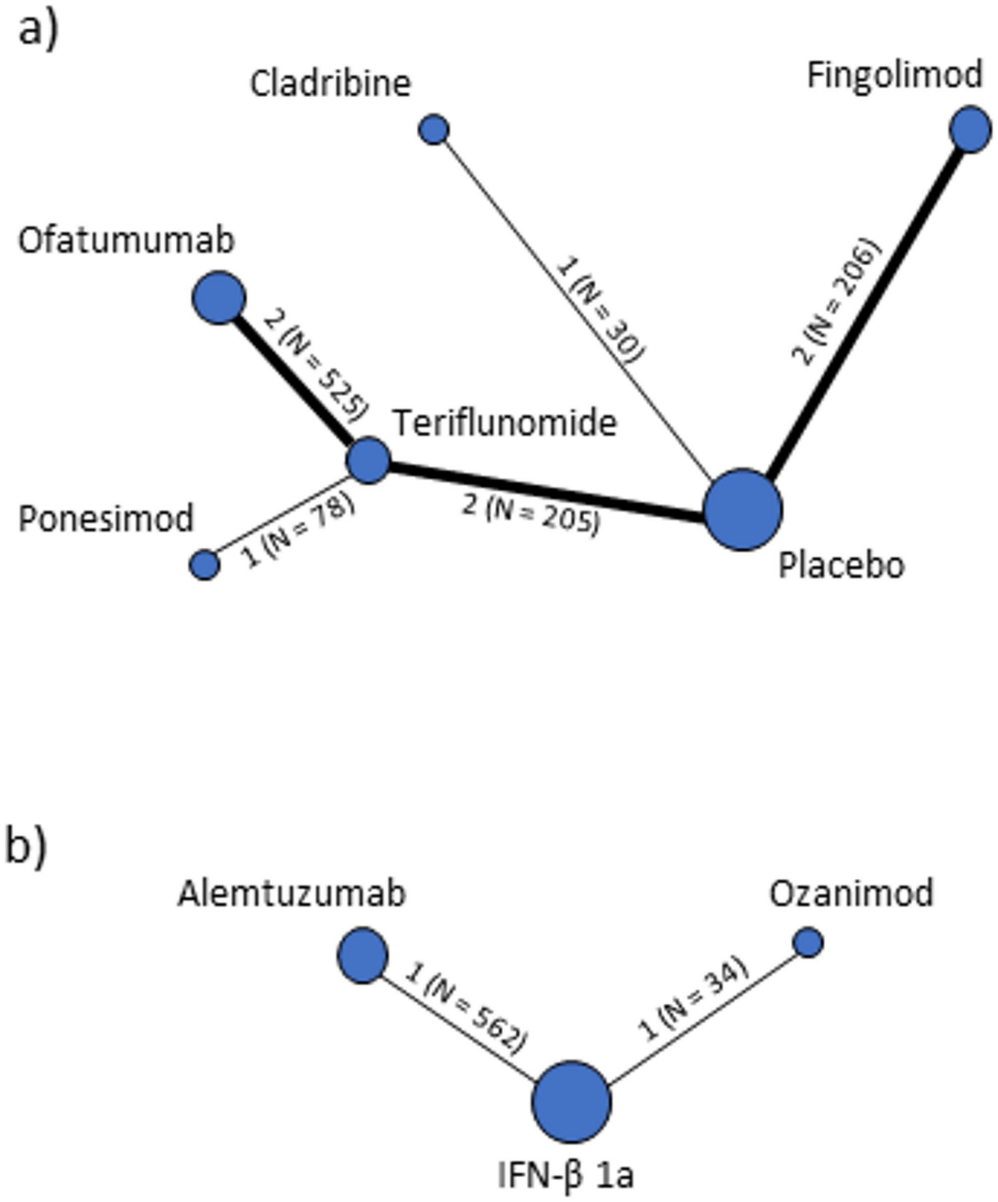
Comparisons for the outcome of annual relapse rate; including all DMTs with available data on subpopulations with highly active RRMS despite previous DMT treatment.Numbers indicate the number of studies per comparison and the total number of patients included. DMT = disease modifying therapy; N = total number of patients; RRMS = relapsing-remitting multiple sclerosis

For the ARR outcome, the relevant studies can be grouped into 2 subnetworks. One subnetwork (Fig. 2a) includes the studies investigating cladribine, fingolimod, ofatumumab, ponesimod and teriflunomide and is grouped around the common comparators placebo and teriflunomide. The other subnetwork (Fig. 2b) consists only of an indirect comparison of alemtuzumab and ozanimod via the common comparator IFN-β 1a. We did not identify any studies that connected the 2 subnetworks. Apart from the 3 studies that compared ofatumumab or ponesimod with teriflunomide, there were no studies that directly compared the DMTs with each other.

### Data for individual outcomes

**Fig. 3:**
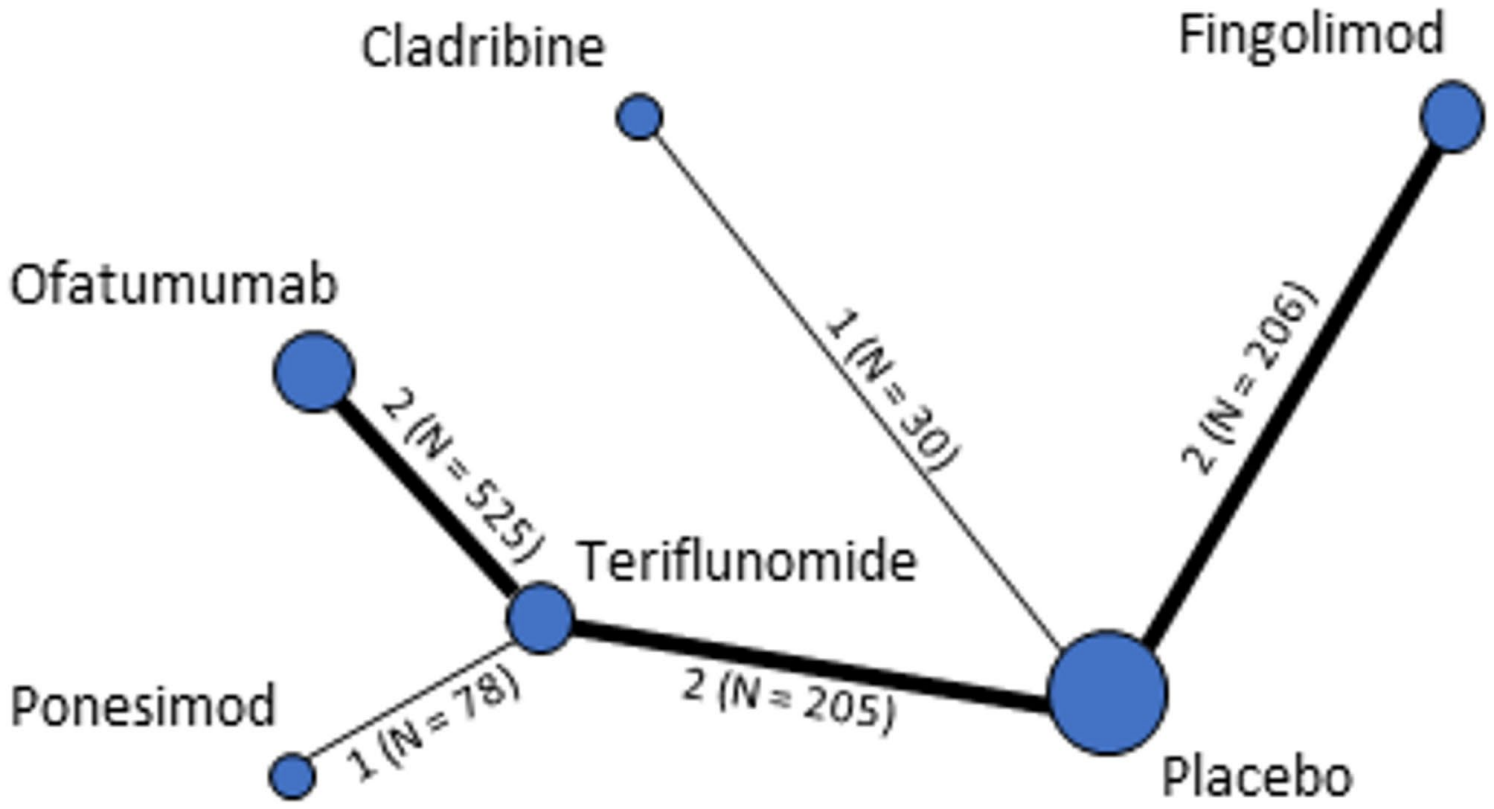
Comparisons for the outcome of serious adverse events; including all DMTs with available data on subpopulations with highly active RRMS despite previous DMT treatment. Numbers indicate the number of studies per comparisonand the total number of patients included. DMT = disease modifying therapy; N = total number of patients; RRMS = relapsing-remitting multiple sclerosis

At the outcome level, the number of studies included ranged from 1 for HRQoL (OPTIMUM study, ponesimod versus teriflunomide) to 10 for ARR (all studies for which subpopulation data were provided; see Supplementary Table 1 for references). Comparisons for specific outcomes are shown in Figs. 2 and 3 and Supplementary Fig. 1. This variation in numbers is due to the varying availability of outcome data in each study. While MS-specific outcomes such as ARR and EDSS-based disability progression were assessed in all studies, other equally important outcomes such as fatigue, disability severity (as measured by the MSFC) and visual impairment were either not assessed in all studies or the results were not comparable. The latter was mostly due to the different types of analyses or instruments used. Supplementary Table 3 provides an overview of the outcomes that were included in the comparative analysis and, if not, the reasons for their exclusion.

### Assessment of the certainty of the NMA results

As mentioned above, we identified only 2 direct comparisons between DMTs (ofatumumab versus teriflunomide and ponesimod versus teriflunomide). No studies were identified that allowed an indirect comparison of these DMTs. Therefore, it was not possible to test the assumption of consistency for any of the comparisons. The similarity of the relevant studies was assessed using the study information and baseline patient characteristics provided by the study sponsors (see Supplementary Tables 4 and 5), and it was concluded that all studies and populations were sufficiently similar to be considered in one analysis. The assumption of homogeneity was tested for all pairwise comparisons involving at least 2 studies. Statistically significant heterogeneity was identified for the teriflunomide versus placebo comparison for 2 outcomes (ARR and treatment discontinuation due to AEs). For the ARR outcome, heterogeneity was resolved by either excluding 1 of the 2 studies from the NMA and examining the 2 resulting analyses for qualitatively different results, which did not occur (see Table 2). For treatment discontinuation due to AEs, the majority of comparisons between DMTs were inconclusive in both analyses due to the low precision of the estimates. Therefore, the comparison between teriflunomide and placebo was excluded from the network. In consequence, only the results of the direct comparisons (ofatumumab versus teriflunomide and ponesimod versus teriflunomide) were considered; comparisons of fingolimod with other DMTs were not possible for this outcome.

**Table 2:** NMA results for ARR (rate ratio) and SAE (relative risk) from analyses in patients with highly active RRMS despite previous DMT

Comparison of DMTs (horizontal vs. vertical)	Alemtuzumab	Cladribine	Dimethyl fumarate	Fingolimod	Natalizumab	Ocrelizumab	Ofatumumab	Ozanimod	Ponesimod	Teriflunomide
Alemtuzumab		–	–	–	–	x	–	–	–	–
Cladribine	–		x	–	–	–	–	–	–	–
Dimethyl fumarate	–	x		x	–	–	x	–	x	x
Fingolimod	–	–	x		–	–	ARR^a^: 1.03 [0.45; 2.39] 2.41 [0.98; 5.93] SAE: 1.83 [0.60; 5.60]	–	–	ARR:– SAE: 1.61 [0.60; 4.29]
Natalizumab	–	–	–	–		–	–	–	–	–
Ocrelizumab	x	–	–	–	–		–	x	–	–
Ofatumumab	–	–	x	ARR^a^: 0.97 [0.42; 2.24] 0.41 [0.17; 1.02] SAE: 0.55 [0.18; 1.68]	–	–		–	–	**ARR: 0.46 [0.31; 0.68]** SAE: 0.88 [0.51; 1.51]
**Ozanimod**	–	–	–	–	–	x	–		–	–
**Ponesimod**	–	–	x	–	–	–	–	–		**ARR: 0.45 [0.21; 0.97]** SAE: 1.36 [0.43; 4.33]
**Teriflunomide**	–	–	x	ARR:– SAE: 0.62 [0.23; 1.66]	–	–	**ARR: 2.18 [1.48; 3.21]** SAE: 1.14 [0.66; 1.95]	–	**ARR: 2.22 [1.03; 4.81]** SAE: 0.73 [0.23; 2.33]	

*ARR* annual relapse rate, *DMT *disease modifying therapy, *NMA *network meta-analysis, *RRMS *relapsing-remitting multiple sclerosis, *SAE *serious adverse event

Entries printed in bold indicate statistically significant effects (*p* < 0.05)

Based on the available studies, either no comparative analysis is possible, or results are not interpretable, because only one study with a high risk of bias on at least one comparison of an indirect comparison was available

x: Data for the subpopulation of patients with highly active RRMS despite previous DMT were not provided for at least one of the DMTs

a. Due to significant heterogeneity for the comparison between teriflunomide and placebo, the analysis was run twice, excluding either the study TEMSO or TOWER. The first result in the cell represents the NMA results under exclusion of TEMSO, the second result represents the NMA results under exclusion of TOWER

Most of the DMTs in the network could only be compared using indirect comparisons, which by their nature provide limited certainty of evidence.

### Assessment of risk of bias

Across outcomes, the risk of bias was low for all studies with subpopulation data. However, for individual outcomes, the risk of bias was high for the results of all outcomes in all studies except RADIANCE B (ozanimod versus IFN-β 1a) and OPTIMUM (ponesimod versus teriflunomide). RADIANCE B had a low risk of bias for all outcomes except for disability severity and walking ability, whereas OPTIMUM had a high risk of bias for all outcomes except for disability severity and walking ability.

### Comparative effects of DMTs

Table 2 shows the NMA results for the outcomes ARR and SAEs, as these were assessed in all studies and comparable results were available for most DMTs (see Figs. 2 and 3). However, no SAEs occurred in the RADIANCE B study (ozanimod versus IFN-β 1a). Therefore, a quantitative assessment of the effect between alemtuzumab and ozanimod was not performed for this outcome. As shown in Table 2, for most of the intended comparisons no relevant data were available, or only one study with a high risk of bias was available for at least one comparison of an indirect comparison. In the latter case, the available evidence was not sufficient to answer our research question. Looking at the interpretable results, there were statistically significant differences between DMTs for which only direct comparisons were available. For the ARR outcome, there were statistically significant differences in favour of ofatumumab versus teriflunomide and ponesimod versus teriflunomide. There were no statistically significant differences in the number of patients with SAEs.

For other outcomes, the interpretable results for all comparisons are summarised in Supplementary Tables 6, 7 and 8. For most outcomes, comparisons across all DMTs were not possible due to limited data (see Fig. 2 and Supplementary Fig. 1). Statistically significant differences between DMTs for outcomes other than ARR were shown for disability progression (in favour of ofatumumab versus teriflunomide) and treatment discontinuation due to AEs (once in favour of ofatumumab versus teriflunomide and once in favour of teriflunomide versus ponesimod).

## Discussion

### Main findings

The main finding of our systematic review of 10 newer DMTs (alemtuzumab, cladribine, dimethyl fumarate, fingolimod, natalizumab, ocrelizumab, ofatumumab, ozanimod, ponesimod, and teriflunomide) for patients with highly active RRMS despite previous DMT is that there is a lack of comparative data to allow comprehensive NMAs. Due to the limited number of analyses available, no robust conclusions can be drawn from these results obtained from the NMA. We identified only 2 direct comparisons: ofatumumab versus teriflunomide and ponesimod versus teriflunomide. In the first comparison, ofatumumab was more effective than teriflunomide. The results of the second comparison were inconclusive because ponesimod showed better relapse control than teriflunomide but caused more discontinuations due to AEs.

### Comparison with other systematic reviews

To our knowledge, our analysis is the first systematic review in the highly active disease population to include the 10 newer DMTs listed above. Several other systematic reviews comparing DMTs in MS/RRMS have been published, but these either only included a limited number of newer DMTs or included patients regardless of disease activity and previous treatment [20–25]. These reviews were also not limited to the DMTs mentioned above, but also included studies comparing other DMTs such as interferons, glatiramer acetate, rituximab and mitoxantrone with each other or with placebo. In addition, detailed assessments of similarity between the included studies were usually not reported, and it is unclear to what extent they were carried out. Therefore, detailed comparisons of our review with other systematic reviews are not meaningful because of the differences in the patient populations and treatments.

A meta-review published by Sormani et al. [23] reported methodological differences between 27 mixed treatment comparisons (MTCs) published between 2010 and 2019. The authors concluded that the MTCs reviewed differed in important aspects, in particular the populations and the number of DMTs included. They identified only 2 MTCs that focused on patients with highly active and/or rapidly progressing RRMS, and only one of these was a systematic review. This review by Huisman et al. [21] compared fingolimod with natalizumab and dimethyl fumarate. However, the authors included patients regardless of previous treatment, as in the AFFIRM study (natalizumab versus interferon).

### Strengths and limitations of the review

As mentioned above, the validity of NMAs is strongly dependent on sufficient similarity and homogeneity of the included studies [57]. However, the assessment of these characteristics may be limited when systematic reviews have to rely on journal publications and study registries as their main data sources. Both often provide insufficient information on study characteristics and patient populations [58, 59]. For our NMA, however, we had access to the CSRs of all relevant studies. These comprehensive documents include study protocols and complete data on all items collected in a study [60] and are particularly helpful when a detailed description of studies and patients is required.

In addition, for 10 of the 14 identified studies, the sponsors provided patient characteristics and analyses for patients with high disease activity despite previous treatment with interferons, glatiramer acetate, dimethyl fumarate or teriflunomide. This was of paramount importance for our analyses because all relevant studies were designed for approval in a broader MS population, including treatment-naïve patients and those with active, but not necessarily highly active, disease. These subpopulation analyses were only possible because the sponsors provided access to unpublished IPD. This highlights the value of IPD for secondary analyses of studies and supports the call for widespread availability of CSRs and anonymised IPD to maximise the evidence base for clinical research [61, 62].

The availability of CSRs and the re-analysis based on data from primary studies is a major strength of our review. Another strength is the inclusion of patients who met predefined criteria for high disease activity. In the absence of a widely accepted definition of high disease activity in RRMS, we defined a set of criteria that included only patients whose disease characteristics were sufficiently similar across studies. The criteria were chosen to cover the range of clinical manifestations of high disease activity as widely as possible. However, the full set of criteria could not be applied to any of the relevant studies because the data needed to select patients accordingly (e.g. MRI data before study entry or data on relapses causing functional impairment before study entry) were not collected in the studies. Instead, the definition of the target population varied between study sponsors, which meant that it was not possible to examine different subgroups of high disease activity (e.g. clinical vs. MRI-based) that were pre-specified in the protocol of our analysis. Most patients in the target population had been diagnosed with RRMS several years prior to entering the studies, and had all undergone pre-treatment with at least one DMT. However, as the studies began between 2004 and 2016, and patients were diagnosed using different versions of the McDonald criteria (the 2005 or 2010 versions, except in one case) [27], it is not possible to rule out differences in patient characteristics entirely.

Treatment strategies and definitions of disease characteristics have changed since the start of our systematic review. On the one hand, disability progression in RRMS is no longer considered to be exclusively related to relapses [63]. More importantly, treatment of RRMS with newer, more effective DMTs from the time of diagnosis is increasingly considered preferable to initial treatment with interferons or glatiramer acetate [64, 65]. The treatment setting on which our patient selection is based may therefore become less common. However, this does not change the need for both a definition of high disease activity and studies that include this population, as the effectiveness of DMTs may still differ according to disease activity, regardless of the initial treatment.

### Strengths and limitations of the evidence base

Due to our access to CSRs and the additional analyses of our target population, we are confident that we had the most comprehensive evidence base possible for highly active disease despite previous DMT, except for the data not provided by the MAHs. However, there is a general lack of RCTs, and those that are available often show a number of shortcomings:

We identified a maximum of only 2 relevant RCTs for each DMT, and for half of them only one. All of these RCTs were pivotal studies designed for and completed before approval. There were no relevant post-approval studies despite the fact that the first approvals in Europe were granted in 2006 (natalizumab) and 2011 (fingolimod). Because of the small number of direct comparisons, most analyses were based on indirect evidence. Indirect comparisons by design have a lower certainty of evidence than direct comparisons, even if the assumptions of similarity and homogeneity are met. Here, the ability to assess the consistency within the NMAs is limited. Indirect comparisons require greater certainty of evidence on both sides of the comparison to provide interpretable results, i.e. results that require at least 2 studies (or on 1 study with a low risk of bias) on each side of the comparison. As Table 2 shows, this condition was fulfilled only in 2 comparisons (fingolimod vs. teriflunomide and fingolimod vs. ofatumumab).

We did not identify any RCTs that specifically included our target population. In all relevant studies, the inclusion criteria allowed recruitment of patients across the entire spectrum of active RRMS. In addition, all studies except one allowed for the inclusion of both treatment-naïve and previously treated patients. We therefore asked the MAHs to provide us with analyses that included only our target population. This population was small in most of the studies. Both the small sample size and the limited number of direct comparative studies diminish the strength of the NMAs.

Unfortunately, the MAHs did not provide subpopulation data for dimethyl fumarate and ocrelizumab. However, as these would have been linked to other DMTs in the network by the same common comparators available from other studies, this data gap did not prevent the other DMTs from being compared with each other. More importantly, the missing subpopulations represented only a small percentage of the total population. The most striking evidence gap was found for natalizumab. In the EU, this DMT is only approved as monotherapy for the treatment of patients with highly active disease despite previous DMT due to safety concerns [11, 66]. We did not identify a single RCT that included the relevant subpopulation to support its approval. The available pivotal studies, AFFIRM and SENTINEL, were conducted in different treatment settings, with AFFIRM limited to treatment-naïve patients [54] and SENTINEL evaluating natalizumab in combination with IFN-β 1a only [55]. Post-approval studies were also lacking.

For people with highly active disease, one concern is that more severe relapses may accelerate disease progression. Furthermore, progression independent of relapse activity (PIRA) is increasingly recognised as an important driver of MS progression [63, 67]. Although the severity of relapses or PIRA was not examined as an outcome in any of the studies, progression-related outcomes were available to capture this aspect, most notably confirmed disability progression (EDSS-based) and severity of disability (MSFC-based).

In line with previous research [68], our evidence base was also limited by a lack of data on patient-relevant outcomes, including patient-reported outcomes. Outcomes such as fatigue and visual impairment were not regularly assessed in the pivotal studies. HRQoL was a prespecified outcome in some studies, but still could not be assessed in our NMA, mainly because either data on subpopulations were not provided or there was a high proportion of missing values, which precluded analyses. For some outcomes, such as specific AEs, few data were provided by the MAHs.

In addition, we did not find any long-term RCTs (> 2 years). This lack of long-term data was also noted by Gerardi et al. [69], who summarised the evidence from pre- and post-approval studies in MS available up to 2017, including studies of alemtuzumab, dimethyl fumarate, fingolimod, natalizumab and teriflunomide. The authors found that only 2 of the 16 pivotal studies followed patients for more than 2 years. As some DMTs are associated with delayed, serious adverse effects, a comprehensive assessment of their benefits and harms requires comparative studies with other DMTs with much longer follow-up periods than the current pivotal studies.

The need for additional post-hoc analyses in all relevant studies in RRMS indicates a large research gap in this therapeutic indication. The restriction of some of the newer DMTs for high disease activity requires comparative studies in these patients. While it is not surprising that these were not designed prior to approval, the general lack of subsequent research in this area leaves both clinicians and patients without important information for their specific needs.

### Future research

The shortcomings described above indicate that there is a need for extensive but targeted research that goes beyond regulatory requirements. Studies designed for this purpose should provide a high certainty of evidence and meet the needs of routine care. One possible approach to meeting these needs is to conduct randomised registry-based trials [70–73] Disease-specific registries can collect and document data from large cohorts of patients, including pre-treated patients and patients with high disease activity. The registry can be used to identify, recruit and randomise a sufficient number of patients. Data collection can use the existing infrastructure of the registry, provided that the quality of the data is sufficient for the intended study, thus saving costs compared to a conventional RCT. In addition, the duration of data collection in a registry is not limited, so patient cohorts can be followed for several years. In any case, randomised registry-based trials are currently being discussed as a superior alternative to non-randomised trials because they combine feasibility, pragmatism and a high certainty of evidence. One example of such a study in MS was conducted in a Swedish registry, comparing the effectiveness of rituximab and dimethyl fumarate in preventing relapses in early RRMS [74].

Finally, the issue of de-escalation in patients whose MS has become inactive has recently received more attention [75–77], partly because disease activity may decrease with age, while the risk of infection and other AEs increases [78]. The importance of de-escalation was also emphasised by the patients who attended the project meeting at IQWiG. We therefore aimed to include studies that compared de-escalation with continuing treatment, but could not identify any. Although research has been done in this area, the RCTs we identified did not primarily investigate de-escalation in patients receiving the 10 newer DMTs included in our review, but also in patients receiving first-generation DMTs such as interferons and glatiramer acetate [79–81].

### Conclusions and implications

Our analysis shows that there is very limited evidence to assess the comparative effectiveness of newer DMTs in patients with high disease activity in RRMS despite previous treatment. A conclusive comparison of available DMTs using NMAs would require comprehensive and well-connected networks. The main barriers to meaningful comparisons are the lack of studies specifically designed for this MS subpopulation, especially RCTs that directly compare DMTs, the overall small number of studies per DMT, as well as post-approval studies and studies with long-term follow-up. These findings call for research programmes that promote studies tailored to the above needs, beyond those required for regulatory approval.

## Supplementary Information

Supplementary Material 1

## Data Availability

The data supporting the conclusions of this article are available in the full (German-language) report on the IQWiG website (27). The results of our study were made available to patients and the general public through the publication of the HTA on IQWiG’s website. An easy-to-understand summary will be made available to patients and the general public on IQWiG’s patient information website (English version: https://www.informedhealth.org/).

## Abbreviations

AE: Adverse event
CSR: Clinical study report
DMT: Disease-modifying therapy
EDSS: Expanded Disability Status Scale
EMA: European Medicines Agency
EU: European Union
Gd+: Gadolinium-enhancing
G-BA: Federal Joint Committee
HRQoL: Health-related quality of life
HTA: Health technology assessment
IPD: Individual patient data
IQWiG: Institute for Quality and Efficiency in Health Care
MAH: Marketing authorisation holder
MRI: Magnetic resonance imaging
MS: Multiple sclerosis
MSFC: Multiple Sclerosis Functional Composite
MTC: Mixed treatment comparison
NMA: Network meta-analysis
OECD: Organisation for Economic Co-operation and Development
RCT: Randomised controlled trial
RRMS: Relapsing-remitting multiple sclerosis
SAE: Serious adverse event

## References

[1] Filippi M, Bar-Or A, Piehl F, Preziosa P, Solari A, Vukusic S, Rocca MA. Multiple sclerosis. Nat Reviews Disease Primers. 2018;4(1):43. 30410033 10.1038/s41572-018-0041-4

[2] McGinley MP, Goldschmidt CH, Rae-Grant AD. Diagnosis and treatment of multiple sclerosis: a review. JAMA. 2021;325(8):765–79. 33620411 10.1001/jama.2020.26858

[3] Diaz C, Zarco LA, Rivera DM. Highly active multiple sclerosis: an update. Mult Scler Relat Disord. 2019;30:215–24. 30822617 10.1016/j.msard.2019.01.039

[4] Noseworthy JH, Lucchinetti C, Rodriguez M, Weinshenker BG. Multiple sclerosis. N Engl J Med. 2000;343(13):938–52. 11006371 10.1056/NEJM200009283431307

[5] European Medicines Agency. Ponvory: EPAR - Product Information 2023 [Updated 13.07.2023]. Available from: https://www.ema.europa.eu/en/documents/product-information/ponvory-epar-product-information_en.pdf.

[6] European Medicines Agency. Aubagio: EPAR - Product Information 2023 [Updated 17.08.2023]. Available from: https://www.ema.europa.eu/en/documents/product-information/aubagio-epar-product-information_en.pdf.

[7] European Medicines Agency. Lemtrada: EPAR - Product Information 2023 [Updated 30.10.2023]. Available from: https://www.ema.europa.eu/en/documents/product-information/lemtrada-epar-product-information_en.pdf.

[8] European Medicines Agency. Mavenclad: EPAR - Product Information 2023 [Updated 19.10.2023]. Available from: https://www.ema.europa.eu/en/documents/product-information/mavenclad-epar-product-information_en.pdf.

[9] European Medicines Agency. Tecfidera: EPAR - Product Information 2023 [Updated 05.12.2023]. Available from: https://www.ema.europa.eu/en/documents/product-information/tecfidera-epar-product-information_en.pdf.

[10] European Medicines Agency. Gilenya: EPAR - Product Information 2023 [Updated 15.09.2023]. Available from: https://www.ema.europa.eu/en/documents/product-information/gilenya-epar-product-information_en.pdf.

[11] European Medicines Agency. Tysabri: EPAR - Product Information 2024 [Updated 21.02.2024]. Available from: https://www.ema.europa.eu/en/documents/product-information/tysabri-epar-product-information_en.pdf.

[12] European Medicines Agency. Ocrevus: EPAR - Product Information 2023 [Updated 13.04.2023]. Available from: https://www.ema.europa.eu/en/documents/product-information/ocrevus-epar-product-information_en.pdf.

[13] European Medicines Agency. Kesimpta: EPAR - Product Information 2023 [Updated 01.03.2023]. Available from: https://www.ema.europa.eu/en/documents/product-information/kesimpta-epar-product-information_en.pdf.

[14] European Medicines Agency. Zeposia: EPAR - Product Information 2023 [Updated 03.04.2023]. Available from: https://www.ema.europa.eu/en/documents/product-information/zeposia-epar-product-information_en.pdf.

[15] European Medicines Agency. Summary of risk management plan for LEMTRADA (Alemtuzumab) 2022 [Updated 19.07.2022]. Available from: https://www.ema.europa.eu/en/documents/rmp-summary/lemtrada-epar-risk-management-plan-summary_en.pdf.

[16] European Medicines Agency. PRAC Assessment Report: Procedure under Article 20 of Regulation (EC) No 726/2004 resulting from pharmacovigilance data Procedure number: EMEA/H/A-20/1483/C/3718/0028; Lemtrada 2019 [Updated 31.10.2019]. Available from: https://www.ema.europa.eu/en/documents/referral/lemtrada-article-20-procedure-prac-assessment-report_en.pdf.

[17] European Medicines Agency. Tysabri; EPAR; Scientific Discussion 2007 [Updated 31.05.2007]. Available from: https://www.ema.europa.eu/documents/scientific-discussion/tysabri-epar-scientific-discussion_en.pdf.

[18] European Medicines Agency. Assessment report Mavenclad, International non-proprietary name: cladribine; Procedure No. EMEA/H/C/004230/0000 2017 [Updated 08.09.2017]. Available from: https://www.ema.europa.eu/en/documents/assessment-report/mavenclad-epar-public-assessment-report_en.pdf.

[19] European Medicines Agency. Assessment report Gilenya, International nonproprietary name: Fingolimod; Procedure No. EMEA/H/C/2202 2011 [Updated 17.02.2011]. Available from: https://www.ema.europa.eu/en/documents/assessment-report/gilenya-epar-public-assessment-report_en.pdf.

[20] Fogarty E, Schmitz S, Tubridy N, Walsh C, Barry M. Comparative efficacy of disease-modifying therapies for patients with relapsing remitting multiple sclerosis: systematic review and network meta-analysis. Mult Scler Relat Disord. 2016;9:23–30. 27645339 10.1016/j.msard.2016.06.001

[21] Huisman E, Papadimitropoulou K, Jarrett J, Bending M, Firth Z, Allen F, Adlard N. Systematic literature review and network meta-analysis in highly active relapsing-remitting multiple sclerosis and rapidly evolving severe multiple sclerosis. BMJ Open. 2017;7(3): e013430. 28283486 10.1136/bmjopen-2016-013430 PMC5353339

[22] Li H, Hu F, Zhang Y, Li K. Comparative efficacy and acceptability of disease-modifying therapies in patients with relapsing-remitting multiple sclerosis: a systematic review and network meta-analysis. J Neurol. 2020;267(12):3489–98. 31129710 10.1007/s00415-019-09395-w

[23] Pia Sormani M, Wolff R, Lang S, Duffy S, Hyde R, Kinter E, et al. Overview of differences and similarities of published mixed treatment comparisons on pharmaceutical interventions for multiple sclerosis. Neurol Ther. 2020;9(2):335–58. 32978726 10.1007/s40120-020-00213-4 PMC7606374

[24] Liu Z, Liao Q, Wen H, Zhang Y. Disease modifying therapies in relapsing-remitting multiple sclerosis: a systematic review and network meta-analysis. Autoimmun Rev. 2021;20(6): 102826. 33878488 10.1016/j.autrev.2021.102826

[25] Sladowska K, Kawalec P, Holko P, Osiecka O. Comparative safety of high-efficacy disease-modifying therapies in relapsing-remitting multiple sclerosis: a systematic review and network meta-analysis. Neurol Sci. 2022;43(9):5479–500. 35713731 10.1007/s10072-022-06197-3

[26] Institut für Qualität und Wirtschaftlichkeit im Gesundheitswesen. Alemtuzumab, Cladribin, Dimethylfumarat, Fingolimod, Natalizumab, Ocrelizumab, Ofatumumab, Ozanimod, Ponesimod und Teriflunomid zur Behandlung Erwachsener mit hochaktiver schubförmig remittierender multipler Sklerose; Berichtsplan [Berichtsplan]. 2021 [Updated 13.12.2021]; cited A20-60. Report plan. Available from: https://www.iqwig.de/download/a20-60_multiple-sklerose_berichtsplan_v2-1.pdf.

[27] Institut für Qualität und Wirtschaftlichkeit im Gesundheitswesen. Alemtuzumab, Cladribin, Dimethylfumarat, Fingolimod, Natalizumab, Ocrelizumab, Ofatumumab, Ozanimod, Ponesimod und Teriflunomid zur Behandlung Erwachsener mit hochaktiver schubförmig remittierender multipler Sklerose; Abschlussbericht [Abschlussbericht/ -dokument]. 2023 [Updated 01.07.2024]; cited A20-60. Final report/document; 1625:[Available from: https://www.iqwig.de/download/a20-60_multiple-sklerose_abschlussbericht_v1-0.pdf.

[28] Institute for Quality and Efficiency in Health Care. Alemtuzumab, cladribine, dimethyl fumarate, fingolimod, natalizumab, ocrelizumab, ofatumumab, ozanimod, ponesimod, and teriflunomide for the treatment of adult patients with highly active relapsing remitting multiple sclerosis; English summary 2024 [Updated 24.01.2024]. Available from: https://www.iqwig.de/download/a20-60_multiple-sclerosis_extract-of-final-report_v1-0.pdf. 38386940

[29] Institute for Quality and Efficiency in Health Care. General Methods; Version 7.0 [Abschlussbericht/ -dokument]. 2023 [Updated 19.09.2023]; cited Methoden. Final report/document. Available from: https://www.iqwig.de/methoden/general-methods_version-7-0.pdf.

[30] Hutton B, Salanti G, Caldwell DM, Chaimani A, Schmid CH, Cameron C, et al. The PRISMA extension statement for reporting of systematic reviews incorporating network meta-analyses of health care interventions: checklist and explanations. Ann Intern Med. 2015;162(11):777–84. 26030634 10.7326/M14-2385

[31] Hemmer B. Diagnose und Therapie der Multiplen Sklerose, Neuromyelitis-optica-Spektrum-Erkrankungen und MOG-IgG-assoziierten Erkrankungen, S2k-Leitlinie, 2023 2023 [Updated 30.11.2023]. Available from: https://register.awmf.org/assets/guidelines/030-050l_S2k_Diagnose-Therapie-Multiple-Sklerose-Neuromyelitis-Optica-Spektrum-MOG-IgG-assoziierte-Erkrankungen_2023-05.pdf.

[32] European Medicines Agency. Guideline on clinical investigation of medicinal products for the treatment of Multiple Sclerosis 2015 [Updated 26.03.2015]. Available from: https://www.ema.europa.eu/documents/scientific-guideline/guideline-clinical-investigation-medicinal-products-treatment-multiple-sclerosis_en-0.pdf.

[33] Montalban X, Gold R, Thompson AJ, Otero-Romero S, Amato MP, Chandraratna D, et al. Ectrims/EAN guideline on the pharmacological treatment of people with multiple sclerosis. Eur J Neurol. 2018;25(2):215–37. 29352526 10.1111/ene.13536

[34] National Institute for Health and Care Excellence. Multiple sclerosis in adults: management 2022 [Updated 22.06.2022]. Available from: https://www.nice.org.uk/guidance/ng220. 36279391

[35] American Academy of Neurology. Practice Guideline Recommendations: Disease-modifying Therapies for Adults with Multiple Sclerosis 2018 [Updated 06.03.2018]. Available from: https://www.aan.com/Guidelines/home/GuidelineDetail/898.

[36] England NHS. Treatment Algorithm for Multiple Sclerosis Disease-Modifying Therapies 2024 [Updated 20.06.2023]. Available from: https://www.england.nhs.uk/wp-content/uploads/2024/03/treatment-algorithm-for-multiple-sclerosis-disease-modifying-therapies-july-23.pdf.

[37] Freedman MS, Devonshire V, Duquette P, Giacomini PS, Giuliani F, Levin MC, et al. Treatment optimization in multiple sclerosis: Canadian MS working group recommendations. Can J Neurol Sci. 2020;47(4):437–55. 32654681 10.1017/cjn.2020.66

[38] Ellenberger D, Flachenecker P, Fneish F, Frahm N, Hellwig K, Paul F, et al. Aggressive multiple sclerosis: a matter of measurement and timing. Brain. 2020;143(11):e97. 33175163 10.1093/brain/awaa306 PMC7719018

[39] Iacobaeus E, Arrambide G, Pia Amato M, Derfuss T, Vukusic S, Hemmer B, et al. Aggressive multiple sclerosis (1): towards a definition of the phenotype. Mult Scler. 2020;26(9):1031–44. 10.1177/1352458520925369 PMC7412876 32530385

[40] Fergusson D, Aaron SD, Guyatt G, Hebert P. Post-randomisation exclusions: the intention to treat principle and excluding patients from analysis. BMJ. 2002;325(7365):652–4. 12242181 10.1136/bmj.325.7365.652 PMC1124168

[41] Boutron I, Estellat C, Guittet L, Dechartres A, Sackett DL, Hrobjartsson A, Ravaud P. Methods of blinding in reports of randomized controlled trials assessing pharmacologic treatments: a systematic review. PLoS Med. 2006;3(10): e425. 17076559 10.1371/journal.pmed.0030425 PMC1626553

[42] Schulz KF, Chalmers I, Hayes RJ, Altman DG. Empirical evidence of bias. Dimensions of methodological quality associated with estimates of treatment effects in controlled trials. JAMA. 1995;273(5):408–12. 7823387 10.1001/jama.273.5.408

[43] Schulz KF, Grimes DA. Generation of allocation sequences in randomised trials: chance, not choice. Lancet. 2002;359(9305):515–9. 11853818 10.1016/S0140-6736(02)07683-3

[44] McGauran N, Wieseler B, Kreis J, Schuler YB, Kolsch H, Kaiser T. Reporting bias in medical research - a narrative review. Trials. 2010;11: 37. 20388211 10.1186/1745-6215-11-37 PMC2867979

[45] Kirkham JJ, Dwan KM, Altman DG, Gamble C, Dodd S, Smyth R, Williamson PR. The impact of outcome reporting bias in randomised controlled trials on a cohort of systematic reviews. BMJ. 2010;340: c365. 20156912 10.1136/bmj.c365

[46] Altman DG. Missing outcomes in randomized trials: addressing the dilemma. Open medicine: a peer-reviewed, independent. open-access J. 2009;3(2):e51–3. PMC2765768 19946393

[47] Sterne JAC, Savovic J, Page MJ, Elbers RG, Blencowe NS, Boutron I, et al. RoB 2: a revised tool for assessing risk of bias in randomised trials. BMJ (Clinical Res ed). 2019;366:l4898. 10.1136/bmj.l4898 31462531

[48] Unnebrink K, Windeler J. Intention-to-treat: methods for dealing with missing values in clinical trials of progressively deteriorating diseases. Stat Med. 2001;20(24):3931–46. 11782044 10.1002/sim.1149

[49] Williamson PR, Gamble C, Altman DG, Hutton JL. Outcome selection bias in meta-analysis. Stat Methods Med Res. 2005;14(5):515–24. 16248351 10.1191/0962280205sm415oa

[50] Bucher HC, Guyatt GH, Griffith LE, Walter SD. The results of direct and indirect treatment comparisons in meta-analysis of randomized controlled trials. J Clin Epidemiol. 1997;50(6):683–91. 9250266 10.1016/s0895-4356(97)00049-8

[51] Dias S, Welton NJ, Caldwell DM, Ades AE. Checking consistency in mixed treatment comparison meta-analysis. Stat Med. 2010;29(7–8):932–44. 20213715 10.1002/sim.3767

[52] Institut für Qualität und Wirtschaftlichkeit im Gesundheitswesen. Alemtuzumab, Cladribin, Dimethylfumarat, Fingolimod, Natalizumab, Ocrelizumab, Ofatumumab, Ozanimod, Ponesimod und Teriflunomid zur Behandlung Erwachsener mit hochaktiver schubförmig remittierender multipler Sklerose; Dokumentation der Anhörung zum Berichtsplan 2021 [Updated 12.08.2021]. Available from: https://www.iqwig.de/download/a20-60_multiple-sklerose_da-berichtsplan_v1-0.pdf.

[53] Institut für Qualität und Wirtschaftlichkeit im Gesundheitswesen. Alemtuzumab, Cladribin, Dimethylfumarat, Fingolimod, Natalizumab, Ocrelizumab, Ofatumumab, Ozanimod, Ponesimod und Teriflunomid zur Behandlung Erwachsener mit hochaktiver schubförmig remittierender multipler Sklerose; Dokumentation der Anhörung zum Vorbericht [Dokumentation der Anhörung]. 2023 [Updated 30.08.2023]; cited A20-60. Documentation of the hearing. Available from: https://www.iqwig.de/download/a20-60_multiple-sklerose_da-vorbericht_v1-0.pdf.

[54] Polman CH, O’Connor PW, Havrdova E, Hutchinson M, Kappos L, Miller DH, et al. A randomized, placebo-controlled trial of natalizumab for relapsing multiple sclerosis. N Engl J Med. 2006;354(9):899–910. 16510744 10.1056/NEJMoa044397

[55] Rudick RA, Stuart WH, Calabresi PA, Confavreux C, Galetta SL, Radue EW, et al. Natalizumab plus interferon beta-1a for relapsing multiple sclerosis. N Engl J Med. 2006;354(9):911–23. 16510745 10.1056/NEJMoa044396

[56] Butzkueven H, Licata S, Jeffery D, Arnold DL, Filippi M, Geurts JJ, et al. Natalizumab versus fingolimod for patients with active relapsing-remitting multiple sclerosis: results from REVEAL, a prospective, randomised head-to-head study. BMJ Open. 2020;10(10):e038861. 33082194 10.1136/bmjopen-2020-038861 PMC7577060

[57] Donegan S, Williamson P, D’Alessandro U, Tudur Smith C. Assessing key assumptions of network meta-analysis: a review of methods. Res Synthesis Methods. 2013;4(4):291–323. 10.1002/jrsm.1085 26053945

[58] Wieseler B, Kerekes MF, Vervoelgyi V, McGauran N, Kaiser T. Impact of document type on reporting quality of clinical drug trials: a comparison of registry reports, clinical study reports, and journal publications. BMJ. 2012;344: d8141. 22214759 10.1136/bmj.d8141

[59] Kohler M, Haag S, Biester K, Brockhaus AC, McGauran N, Grouven U, et al. Information on new drugs at market entry: retrospective analysis of health technology assessment reports versus regulatory reports, journal publications, and registry reports. BMJ. 2015;350: h796. 25722024 10.1136/bmj.h796 PMC4353284

[60] ICH. Structure and Content of Clinical Study Reports 1995 [Updated 30.11.1995]. Available from: https://database.ich.org/sites/default/files/E3_Guideline.pdf.

[61] Bierer BE, Li R, Barnes M, Sim IA, Global. Neutral platform for sharing trial data. N Engl J Med. 2016;374(25):2411–3. 27168194 10.1056/NEJMp1605348

[62] Taichman DB, Backus J, Baethge C, Bauchner H, de Leeuw PW, Drazen JM, et al. Sharing clinical trial data–a proposal from the international committee of medical journal editors. N Engl J Med. 2016;374(4):384–6. 26786954 10.1056/NEJMe1515172

[63] Lublin FD, Haring DA, Ganjgahi H, Ocampo A, Hatami F, Cuklina J, et al. How patients with multiple sclerosis acquire disability. Brain. 2022;145(9):3147–61. 35104840 10.1093/brain/awac016 PMC9536294

[64] Spelman T, Magyari M, Piehl F, Svenningsson A, Rasmussen PV, Kant M, et al. Treatment escalation vs immediate initiation of highly effective treatment for patients with relapsing-remitting multiple sclerosis: data from 2 different national strategies. JAMA Neurol. 2021;78(10):1197–204. 34398221 10.1001/jamaneurol.2021.2738 PMC8369379

[65] Weideman AM, Tapia-Maltos MA, Johnson K, Greenwood M, Bielekova B. Meta-analysis of the age-dependent efficacy of multiple sclerosis treatments. Front Neurol. 2017;8: 577. 29176956 10.3389/fneur.2017.00577 PMC5686062

[66] European Medicines Agency. Tysabri: EPAR - Scientific Discussion 2007 [Updated 31.05.2007]. Available from: https://www.ema.europa.eu/en/documents/scientific-discussion/tysabri-epar-scientific-discussion_en.pdf.

[67] Kappos L, Wolinsky JS, Giovannoni G, Arnold DL, Wang Q, Bernasconi C, et al. Contribution of relapse-independent progression vs relapse-associated worsening to overall confirmed disability accumulation in typical relapsing multiple sclerosis in a pooled analysis of 2 randomized clinical trials. JAMA Neurol. 2020;77(9):1132–40. 32511687 10.1001/jamaneurol.2020.1568 PMC7281382

[68] Gehr S, Kaiser T, Kreutz R, Ludwig WD, Paul F. Suggestions for improving the design of clinical trials in multiple sclerosis-results of a systematic analysis of completed phase III trials. EPMA J. 2019;10(4):425–36. 31832116 10.1007/s13167-019-00192-z PMC6883016

[69] Gerardi C, Bertele V, Rossi S, Garattini S, Banzi R. Preapproval and postapproval evidence on drugs for multiple sclerosis. Neurology. 2018;90(21):964–73. 10.1212/WNL.0000000000005561. 10.1212/WNL.0000000000005561 29695598

[70] Lauer MS, D’Agostino RB, Sr. The randomized registry trial–the next disruptive technology in clinical research? N Engl J Med. 2013;369(17):1579–81. 23991657 10.1056/NEJMp1310102

[71] Li G, Sajobi TT, Menon BK, Korngut L, Lowerison M, James M, et al. Registry-based randomized controlled trials- what are the advantages, challenges, and areas for future research? J Clin Epidemiol. 2016;80:16–24. 27555082 10.1016/j.jclinepi.2016.08.003

[72] Bowman L, Weidinger F, Albert MA, Fry ETA, Pinto FJ, Clinical Trial Expert G, Forum ESCP. Randomized trials fit for the 21st century. A joint opinion from the European society of cardiology, American heart association, American college of cardiology, and the world heart federation. Eur Heart J. 2022;44(11):931–4. 10.1093/eurheartj/ehac633 PMC10011328 36525339

[73] Woodcock J, LaVange LM. Master protocols to study multiple therapies, multiple diseases, or both. N Engl J Med. 2017;377(1):62–70. 28679092 10.1056/NEJMra1510062

[74] Svenningsson A, Frisell T, Burman J, Salzer J, Fink K, Hallberg S, et al. Safety and efficacy of rituximab versus dimethyl fumarate in patients with relapsing-remitting multiple sclerosis or clinically isolated syndrome in Sweden: a rater-blinded, phase 3, randomised controlled trial. Lancet Neurol. 2022;21(8):693–703. 35841908 10.1016/S1474-4422(22)00209-5

[75] Vollmer BL, Wolf AB, Sillau S, Corboy JR, Alvarez E. Evolution of disease modifying therapy benefits and risks: an argument for de-escalation as a treatment paradigm for patients with multiple sclerosis. Front Neurol. 2021;12: 799138. 35145470 10.3389/fneur.2021.799138 PMC8821102

[76] Prosperini L, Haggiag S, Ruggieri S, Tortorella C, Gasperini C. Stopping disease-modifying treatments in multiple sclerosis: a systematic review and meta-analysis of real-world studies. CNS Drugs. 2023;37(10):915–27. 37740822 10.1007/s40263-023-01038-z

[77] Coerver EME, Fung WH, de Beukelaar J, Bouvy WH, Canta LR, Gerlach OHH, et al. Discontinuation of first-line disease-modifying therapy in patients with stable multiple sclerosis: the DOT-MS randomized clinical trial. JAMA Neurol. 2025;82(2):123–31. 39652340 10.1001/jamaneurol.2024.4164 PMC11811793

[78] Graves JS, Krysko KM, Hua LH, Absinta M, Franklin RJM, Segal BM. Ageing and multiple sclerosis. Lancet Neurol. 2023;22(1):66–77. 36216015 10.1016/S1474-4422(22)00184-3

[79] Corboy JR, Fox RJ, Kister I, Cutter GR, Morgan CJ, Seale R, et al. Risk of new disease activity in patients with multiple sclerosis who continue or discontinue disease-modifying therapies (DISCOMS): a multicentre, randomised, single-blind, phase 4, non-inferiority trial. Lancet Neurol. 2023;22(7):568–77. 37353277 10.1016/S1474-4422(23)00154-0

[80] Rennes University Hospital. Disease Modifying Therapies Withdrawal in Inactive Secondary Progressive Multiple Sclerosis Patients Older Than 50 Years (STOP-I-SEP) 2023 [Updated 24.10.2023]. Available from: https://clinicaltrials.gov/study/NCT03653273.

[81] Amsterdam UMC, location, VUmc. Discontinuing Disease-modifying Therapies in Stable Relapsing - Onset Multiple Sclerosis (DOT-MS) 2020 [Updated 19.10.2020]. Available from: https://clinicaltrials.gov/study/NCT04260711.

