# Supplementary material for: Comparative effectiveness of disease-modifying therapies for highly active relapsing-remitting multiple sclerosis despite previous treatment – a systematic review and network meta-analysis
Source: BMC Neurol. 2025 Aug 9;25:328. doi: 10.1186/s12883-025-04338-7 (PMC12335774; doi:10.1186/s12883-025-04338-7)
Supplement: Supplementary file 1 — Supplementary Material 1 [file 12883_2025_4338_MOESM1_ESM.pdf]

Appendix

1 Information retrieval from marketing authorisation holders (MAHs)

Information about clinical trials on the disease modifying therapies (DMTs) of interest was requested directly from the MAHs. As a prerequisite for the use of unpublished data from MAHs, IQWiG asked the MAHs to sign an agreement requiring the submission of a list of all sponsored published and unpublished studies investigating the DMT concerned, as well as CONSORT compliant documents, in general, the complete clinical study reports (CSRs), on all relevant studies selected by IQWiG. This procedure was required to avoid bias due to selective provision of data. The Common Technical Documents, as submitted to the EMA, were screened for relevant studies. For potentially relevant studies, CSRs were requested from the MAHs.

If a study population was not limited to our target population as outlined above, patient characteristics and study results for the relevant subpopulation were requested from the MAHs. This procedure was also aimed at obtaining patient populations with sufficient similarity across studies and drugs, as the assessment of similarity is an important prerequisite for network meta-analysis (NMA).

2 Data synthesis and statistical methods

The prerequisite for conducting the NMA was an adequate structural quality of the study pool — that is, the availability of a study pool meeting the assumptions of similarity, homogeneity, and consistency. In general, an indirect comparison allows the simultaneous estimation of an effect between two treatments (A and B). This is possible even in the absence of head-to-head trials if other trials comparing A or B with a common comparator C (intermediate comparator)

are available [103,104]. The validity of an indirect comparison requires the assumption of similar study characteristics, such as the study design applied or the population investigated [105]. If this assumption is not met, possible effect modifiers may affect the indirect estimate. A consequence of this assumption is that both the direct and the indirect estimates of the effect of A versus B produce comparable results. While the conceptual assumption is called “similarity” or “transitivity,” the statistical consequence is called “consistency” [106].

While the similarity assumption focuses on similarity of populations, interventions, comparators, outcomes, comparable design, and inclusion and exclusion criteria, the homogeneity assumption relates to treatment effects, as substantial heterogeneity makes a combined statement about the effect difficult. Both are also required in conventional meta-analyses [107-110].

Risk ratios and mean differences were the effect measures used for binary and continuous outcomes, respectively. Risk ratios and their standard errors were normalised by taking the logarithms of the data from contingency tables. In the case of zero events in either treatment group, a factor of 0.5 was added to each cell. Estimates of mean differences and their standard errors were extracted from the original scales used in the primary studies. In the case of statistically significant effects, the final NMA results of a continuous outcome analysis were supported by an additional analysis based on standardised effects using Hedges’ g to assess the relevance of the effect. A relevant difference was assumed if the effect of the NMA based on standardised data was outside the interval (−0.2 to 0.2) [111]. All NMAs were conducted in a frequentist setting using random-effects models [112]. We used the netmeta software package in R [113] (Supplement 3 includes the programming code). Each hypothesis of no

difference between any two treatments was tested locally at the 0.05 level. We did not adjust for multiple testing. The resulting estimates from the NMA are presented with 95% confidence intervals.

We aimed to estimate differences between DMTs quantitatively. We refrained from ranking the NMA results, because our focus was on estimating actual treatment contrasts. In addition, each ranking method responds to a different question of interest and various methodological problems can hamper interpretation [114,115]. For example, ranking methods have been shown to be sensitive to network composition [116], do not reflect a treatment's effect size [117], and cannot always be accompanied by confidence or credible intervals [114]. In contrast to the presentation of effect estimates and confidence or credible intervals, there is no standard recommendation on whether the results should be ranked and, if so, which method should be used.

3 Comments and modifications to the original protocol

After publication of the protocol, an open commenting process was conducted. The MAHs of the DMTs studied, German medical societies with a focus on multiple sclerosis and neurology (German Neurological Society, German Multiple Sclerosis Society, Disease-Related Competence Network Multiple Sclerosis) and patient organisations commented on the protocol. The comments and the resulting changes to the protocol are discussed in detail in IQWiG's final report [118]. These comments led to the publication of an updated protocol. The main changes to the original protocol were the revision of the criteria for high disease activity and the exclusion of studies with a treatment duration of less than approximately 2 years, whereas the original version excluded studies with a duration of less than 1 year. After

publication of the preliminary report, an identical commenting process was initiated, the results of which are also documented in the final report.

4 Programming code for network meta-analyses

```
# Prerequisites: install and load package netmeta,
# if required, load packages required for netmeta are usually automatically loaded.
library(netmeta)


# data input:
# create a data frame nmadata containing
# - study name (StName)
# - treatment effect (md)
# - standard error (se)
# - name of first treatment (T1)
# - name of second treatment/comparator (T2)
# values in brackets are variable names that appear in the following programming statements
# binary data: enter the logarithms of effects and standard error


# network meta-analysis for binary outcome, relative risk:
object.nma.results.bin <- netmeta(te ,se , T1, T2, StName, data=nmadata, sm="RR",
comb.random=TRUE)
# effect measure for binary outcomes: Relative Risk
effect <- "RR"
# effect measure for continuous outcomes : Mean Difference:
```

```
effect <- "MD"


# calculation of network meta-analysis:
nma.net <- netmeta(te, se, B1, B2, StName, data = nmadata,
sm = "effect", level = 0.95, fixed = FALSE)


# results output (relative risk):
exp(nma.net$TE.random)    # treatment effect
exp(nma.net$lower.random) # 95% confidence interval lower bound
exp(nma.net$upper.random) # 95% confidence interval upper bound


# results output (mean difference):
nma.net$TE.random    # treatment effect
nma.net$lower.random # 95% confidence interval lower bound
nma.net$upper.random # 95% confidence interval upper bound
```

Supplementary Table 1 Relevant studies for comparison of escalation therapies in patients with highly active RRMS despite previous DMT

DMT[a]	Study	Publications	Registry	Clinical study report provided (unpublished)	Additional analyses for subpopulation with highly active RRMS despite previous DMT provided [unpublished]
**vs. interferons**					
Alemtuzumab vs. IFN-β 1a	CARE-MS II	[1-5]	[6,7]	yes	yes
Ocrelizumab vs. IFN-β 1a	OPERA I	[8-13]	[14-17]	yes	no
Ocrelizumab vs. IFN-β 1a	OPERA II	[8-13,18]	[19,20]	yes	no
Ozanimod vs. IFN-β 1a	RADIANCE B	[21,22]	[23,24]	yes	yes
**vs. placebo**					
Cladribine vs. placebo	CLARITY	[25-38]	[39,40]	yes	yes
Dimethyl fumarate vs. placebo	CONFIRM	[41-46]	[47-49]	yes	no
Dimethyl fumarate vs. placebo	DEFINE	[42,43,50-54]	[55-58]	yes	no
Fingolimod vs. placebo	FREEDOMS	[42,59-71]	[72,73]	yes	yes
Fingolimod vs. placebo	FREEDOMS II	[62,67,71,74,75]	[76,77]	yes	yes
Teriflunomide vs. placebo	TEMSO	[78-82]	[83,84]	yes	yes
Teriflunomide vs. placebo	TOWER	[42,85-88]	[89-91]	yes	yes
**vs. DMT**					
Ofatumumab vs. teriflunomide	ASCLEPIOS I	[92]	[93-95]	yes	yes
Ofatumumab vs. teriflunomide	ASCLEPIOS II	[92]	[96-99]	yes	yes
Ponesimod vs. teriflunomide	OPTIMUM	[100]	[101,102]	yes	yes

a. No relevant studies investigating natalizumab were identified.

DMT: disease modifying therapy; IFN: Interferon; RRMS: relapsing-remitting multiple sclerosis; vs.: versus

Supplementary Table 2 Definitions of highly active RRMS despite previous DMT as applied by MAHs for additional analyses of study data

**DMT**<br>**Study**	**Definition of adequate and complete previous DMT treatment**	**Highly active RRMS diagnosed after complete course of previous DMT**	**Definition of high disease activity[a]**		
			**Exclusively by clinical criteria**	**By clinical and MRT-based criteria**	**Exclusively by MRT-based criteria**
**Studies comparing DMT vs IFN-β 1a**					
Alemtuzumab vs. IFN-β 1a<br>CARE-MS II	▪ Previous DMT ≥ 3 months (IFN) or ≥ 6 months (GA) within the last year before determination of high disease activity	yes	▪ ≥ 1 relapse in last 12 months or ≥ 2 relapses in last 24 months before randomisation and no Gd+ lesion at baseline<br>▪ Information on severity of relapses not available	≥ 1 relapse in last 12 months and ≥ 1 new Gd+ lesion at baseline	–
Ozanimod vs IFN-β 1a<br>RADIANCE B	▪ Previous DMT ≥ 3 months (IFN) or ≥ 6 months (other DMT)<br>▪ Complete course of therapy within 12 months before baseline	yes	–	≥ 1 relapse in last 12 months and ≥ 1 Gd+ lesion at baseline	–
**Placebo-controlled studies**					
Cladribine vs. placebo<br>CLARITY	▪ Previous DMT ≥ 3 months (IFN) or ≥ 6 months (GA)<br>▪ End of previous DMT within 12 months before baseline	yes	–	≥ 1 relapse in last 12 months and ≥ 1 Gd+ lesion in MRT scan	≥ 9 Gd+ lesions within the last 12 months in MRT scan

Supplementary Table 2 Definitions of highly active RRMS despite previous DMT as applied by MAHs for additional analyses of study data

DMT<br>Study	Definition of adequate and complete previous DMT treatment	Highly active RRMS diagnosed after complete course of previous DMT	Definition of high disease activity[a]		
			Exclusively by clinical criteria	By clinical and MRT-based criteria	Exclusively by MRT-based criteria
Fingolimod vs. placebo<br>FREEDOMS I / FREEDOMS II	▪ Previous DMT of ≥ 90 days or 180 days before relapse<br>▪ End date of previous DMT within 12 months before baseline (if date of relapse available)	yes	▪ ≥ 1 relapse in last 12 months or ≥ 2 relapses in last 24 months before inclusion in study<br>▪ Information on functional impairment not available	–	–
Teriflunomide vs. placebo<br>TEMSO	▪ Previous DMT ≥ 3 months (IFN) or ≥ 6 months (GA) within the last year before determination of high disease activity<br>▪ Last 365 days before screening were considered for duration of treatment	yes	▪ ≥ 1 relapse in last 12 months or ≥ 2 relapses in last 24 months before randomisation and no Gd+ lesion at baseline<br>▪ Information on severity of relapses not available	≥ 1 relapse in last 12 months and ≥ 1 new Gd+ lesion at baseline	–
Teriflunomide vs. placebo<br>TOWER	▪ Previous DMT ≥ 3 months (IFN) or ≥ 6 months (GA) within the last year before determination of high disease activity<br>▪ Last 365 days before screening were considered for duration of treatment	yes	▪ ≥ 1 relapse in last 12 months or ≥ 2 relapses in last 24 months before randomisation<br>▪ Information on severity of relapses not available	–	–

Supplementary Table 2 Definitions of highly active RRMS despite previous DMT as applied by MAHs for additional analyses of study data

DMT<br>Study	Definition of adequate and complete previous DMT treatment	Highly active RRMS diagnosed after complete course of previous DMT	Definition of high disease activity[a]		
			Exclusively by clinical criteria	By clinical and MRT-based criteria	Exclusively by MRT-based criteria
**Studies comparing DMTs directly**					
Ofatumumab vs. Teriflunomide<br>ASCLEPIOS I / ASCLEPIOS II	▪ Previous DMT ≥ 90 days or 180 days before last relapse<br>▪ End date of previous DMT within 12 months before baseline (if date of relapse available)	yes	▪ ≥ 1 relapse in last 12 months or ≥ 2 relapses in last 24 months before inclusion in study<br>▪ Information on functional impairment not available	–	–
Ponesimod vs. teriflunomide<br>OPTIMUM	▪ Previous DMT ≥ 6 months in the year before inclusion into the study; end date 2 months before relapse at most	yes	–	≥ 1 relapse in last year before and ≥ 1 Gd+ lesion at baseline	–

a. If MAHs provided > 1 definition of high disease activity per study, these definitions represent mutually exclusive subpopulations, i. e. patients are counted in only one category; for our analysis we used the combined populations of all patients with highly active RRMS despite previous DMT per study.

DMT: disease modifying therapy; GA: glatiramer acetate; Gd+: gadolinium-enriching; IFN: interferon; MAH: market authorisation holder; MRT: magnetic resonance tomography; RRMS: relapsing-remitting multiple sclerosis

Supplementary Table 3 Patient-relevant outcomes included in the studies and data availability for analyses

DMT	Outcome														
	Overall mortality	Annual relapse rate	Number of patients with confirmed relapse	Confirmed disability progression (EDSS-based), confirmed after 24 weeks	Severity of disability (MSFC-score)	Mobility[a]	Visual impairment	Fatigue	Health-related quality of life	SAE	Discontinuation due to AE	PML	Serious infections	Serious neoplasms	Serious autoimmune disorders
**Versus IFN-β 1a**															
Alemtuzumab	(●)	●	●	●	●	●	x	–	(●)	(●)	(●)[b]	x	x	x	x
Ocrelizumab	x	x	x	x	x	x	x	x	x	x	x	x	x	x	x
Ozanimod	(●)	●	●	●	●	●	(●)	–	(●)	(●)	(●)	(●)	(●)	(●)	–
**Versus placebo**															
Cladribine	(●)	●	●	(●)	–	–	–	–	x	●	(●)[b]	(●)	(●)	(●)	(●)
Dimethyl fumarate	x	x	x	x	x	x	x	–	x	x	x	x	x	x	x
Fingolimod	(●)	●	●	●	(●)	(●)	(●)	–	x	●	(●)[c]	(●)	x	x	x
Teriflunomide	(●)	●	●	●	x	x	–	○	○	●	(●)[c]	x	x	x	x
**Direct comparison**															
Ofatumumab vs. teriflunomide	(●)	●	●	●	●	●	–	–	–	●	●	●	x	x	x
Ponesimod vs. teriflunomide	(●)	●	●	●	●	●	–	○	●	●	●	●	●	●	x
**Natalizumab**	No relevant studies were identified.														

Supplementary Table 3 Patient-relevant outcomes included in the studies and data availability for analyses

DMT	Outcome														
	Overall mortality	Annual relapse rate	Number of patients with confirmed relapse	Confirmed disability progression (EDSS-based), confirmed after 24 weeks	Severity of disability (MSFC-score)	Mobility[a]	Visual impairment	Fatigue	Health-related quality of life	SAE	Discontinuation due to AE	PML	Serious infections	Serious neoplasms	Serious autoimmune disorders

●: Data were provided.

(●): Data were provided, but could not be considered for the analysis, since pairwise comparison between 2 DMTs was not possible (e. g. because no data from studies on other DMTs were available), assumption of homogeneity was violated, or because no events or only few events in 1 study arm occurred

○: Data were provided, but were not suitable for analysis (e. g. because > 30% of the study population were missing from the analysis).

x: Data for the subpopulation of patients with highly active disease despite previous DMT were not provided.

–: Outcome not assessed.

a. Evaluated using the 25-foot walk test.

b. Comparison to other DMTs was inconclusive due to deviating treatment regimens.

c. Consideration of placebo-controlled studies led to imprecise estimations in the NMA; therefore, only direct comparisons of DMTs were considered.

AE: adverse event; DMT: disease modifying therapy; EDSS: Expanded Disability Status Scale; IFN: Interferon; MSFC: Multiple Sclerosis Functional Composite; MSIS: Multiple Sclerosis Impact Scale; NMA: network meta-analysis; PML: progressive multifocal leukoencephalopathy; SAE: serious adverse event

Supplementary Table 4 Patient characteristics of the relevant subpopulations including treatment and study discontinuation

Comparison<br>Study<br>Study arm	N	Age [years] Mean (SD)	Sex [f / m] %	Region n (%)		Previous DMT n (%)				Treatment discontinuation n (%)	Study discontinuation n (%)
				OECD	Non-OECD	IFN-β 1a	IFN-β 1b	Glatiramer acetate	Other DMT[a]		
**Studies comparing DMT vs. IFN-β 1a**											
**Alemtuzumab vs. IFN-β 1a**											
CARE-MS II											
Alemtuzumab	363	35 (9)	65 / 35	283 (78)	80 (22)	155 (43)	99 (27)	85 (23)	n/a [b]	n/a	13 (4)
IFN-β 1a	199	36 (9)	66 / 34	160 (80)	39 (20)	87 (44)	48 (24)	53 (27)	n/a [b]	n/a	44 (22)
**Ocrelizumab vs. IFN-β 1a**											
OPERA I	Data for the relevant subpopulation were not provided										
OPERA II	Data for the relevant subpopulation were not provided										
**Ozanimod vs. IFN-β 1a**											
RADIANCE B											
Ozanimod	17	36 (9)	77 / 24	n/a	n/a	3 (18)	11 (65)	6 (35)	0 (0)[c]	2 (12)[d]	2 (12)[d]
IFN-β 1a	17	33 (7)	71 / 29	n/a	n/a	5 (29)	9 (53)	2 (12)	1 (6)[c]	2 (12)[d]	2 (12)[d]
**Placebo-controlled studies**											
**Cladribine vs. placebo**											
CLARITY											
Cladribine	13	32 (5)	62 / 38	7 (54)	6 (46)	8 (62)[e]	4 (31)[e]	1 (8)[e]	n/a	n/a	4 (31)
Placebo	17	34 (7)	82 / 18	12 (71)	5 (29)	11 (65)[e]	5 (29)[e]	1 (6)[e]	n/a	n/a	3 (18)
**Dimethyl fumarate vs. placebo**											
CONFIRM	Data for the relevant subpopulation were not provided										
DEFINE	Data for the relevant subpopulation were not provided										

Supplementary Table 4 Patient characteristics of the relevant subpopulations including treatment and study discontinuation

Comparison<br>Study<br>Study arm	N	Age [years] Mean (SD)	Sex [f / m] %	Region n (%)		Previous DMT n (%)				Treatment discontinuation n (%)	Study discontinuation n (%)
				OECD	Non-OECD	IFN-β 1a	IFN-β 1b	Glatiramer acetate	Other DMT[a]		
**Fingolimod vs. placebo**											
FREEDOMS											
Fingolimod	34	38 (10)	74 / 27	33 (97)	1 (3)	22 (65)	6 (18)	6 (18)	0 (0)	9 (27)	7 (21)
Placebo	28	39 (8)	79 / 21	27 (96)	1 (4)	16 (57)	6 (21)	6 (21)	0 (0)	10 (36)	8 (29)
FREEDOMS II											
Fingolimod	75	41 (8)	79 / 21	75 (100)	0 (0)	40 (53)	14 (19)	26 (35)	0 (0)	24 (32)	17 (23)
Placebo	69	42 (7)	73 / 28	69 (100)	0 (0)	33 (48)	11 (16)	29 (42)	3 (4)[f]	21 (30)	15 (22)
**Teriflunomide vs. placebo**											
TEMSO											
Teriflunomide	39	38 (7)	72 / 28	37 (95)	2 (5)	29 (74)	6 (15)	5 (13)	n/a	n/a	12 (31)
Placebo	32	37 (9)	84 / 16	30 (94)	2 (6)	21 (66)	3 (9)	7 (22)	n/a	n/a	19 (59)
TOWER											
Teriflunomide	66	39 (10)	73 / 27	61 (92)	5 (8)	37 (56)	15 (23)	18 (27)	n/a	n/a	24 (36)
Placebo	68	37 (10)	72 / 28	58 (85)	10 (15)	29 (43)	15 (22)	26 (38)	n/a	n/a	14 (21)
**Studies comparing DMTs directly**											
**Ofatumumab vs. teriflunomide**											
ASCLEPIOS I											
Ofatumumab	121	39 (8)	64 / 36	88 (73)	33 (27)	29 (24)	17 (14)	39 (32)	39 (32)	n/a	14 (12)
Teriflunomide	122	38 (10)	67 / 33	90 (74)	32 (26)	32 (26)	18 (15)	35 (29)	44 (36)	n/a	22 (18)

Supplementary Table 4 Patient characteristics of the relevant subpopulations including treatment and study discontinuation

Comparison<br>Study<br>Study arm	N	Age [years] Mean (SD)	Sex [f / m] %	Region n (%)		Previous DMT n (%)				Treatment discontinuation n (%)	Study discontinuation n (%)
				OECD	Non-OECD	IFN-β 1a	IFN-β 1b	Glatiramer acetate	Other DMT[a]		
ASCLEPIOS II											
Ofatumumab	135	38 (9)	67 / 33	83 (62)	52 (39)	36 (27)	27 (20)	45 (33)	40 (30)	n/a	18 (13)
Teriflunomide	147	39 (9)	73 / 27	99 (67)	48 (33)	41 (28)	18 (12)	44 (30)	58 (40)	n/a	36 (25)
**Ponesimod vs. teriflunomide**											
OPTIMUM											
Ponesimod	33	36 (9)	61 / 39	19 (58)	14 (42)	n/a	n/a	n/a	n/a	n/a	8 (24)
Teriflunomide	45	37 (9)	58 / 42	28 (62)	17 (38)	n/a	n/a	n/a	n/a	n/a	9 (20)
**Natalizumab**	No relevant studies were identified.										

a. Alemtuzumab, cladribine, dimethyl fumarate, fingolimod, natalizumab, ocrelizumab, ofatumumab, ozanimod, ponesimod, teriflunomide.
b. Other DMTs were used in < 1% of patients.
c. Teriflunomide.
d. Authors' own calculation.
e. No data on time frame for assessment before baseline.
f. Natalizumab.

DMT: disease modifying therapy; f: female; IFN: interferon; m: male; N: number of randomised patients; n: number of patients with event; n/a: not available; OECD: Organisation for Economic Co-operation and Development; SD: standard deviation; vs.: versus

Supplementary Table 5 Disease-specific characteristics of the relevant subpopulations at baseline

Comparison<br>Study<br>Study arm	N	EDSS at baseline<br>Mean (SD)	Time since diagnosis of MS [years]<br>Mean (SD)	Nunber of relapses before baseline<br>Median [Q1; Q3] or (Min; Max)		Number of lesions at baseline<br>Median [Q1; Q3] or (Min; Max)		
				Within 1 year	Within 2 years	T1 Gd+	T1 Hypointense	T2
**Studies comparing DMT vs IFN-β 1a**								
**Alemtuzumab vs. IFN-β 1a**								
CARE-MS II								
Alemtuzumab	363	2.6 (1.2)	4.3 (2.6)	2.0 (0; 5)	2,0 (1; 9)	0 (0; 36)	n/a	n/a
IFN-β 1a	199	2.7 (1.2)	4.4 (2.7)	1.0 (0; 5)	2.0 (1; 7)	0 (0; 41)	n/a	n/a
**Ocrelizumab vs. IFN-β 1a**								
OPERA I				Data for the relevant subpopulation were not provided				
OPERA II				Data for the relevant subpopulation were not provided				
**Ozanimod vs. IFN-β 1a**								
RADIANCE B								
Ozanimod	17	2.6 (1.2)	5.8 (4.0)	1.0 (0.0; 2.0)	2.0 (1.0; 4.0)	2 (1; 13)	n/a	59 (36; 183)
IFN-β 1a	17	2.6 (1.1)	5.9 (4.2)	1.0 (0.0; 3.0)	2.0 (1.0; 4.0)	2 (1; 22)	n/a	53 (33; 145)
**Placebo-controlled studies**								
**Cladribine vs. placebo**								
CLARITY								
Cladribine	13	3.0 (1.7)	7.2 (4.4)	2.0 [1.0; 2.0]	n/a	1 [1; 2]	4 [2; 7]	26 [20; 32]
Placebo	17	2.7 (1.2)	8.3 (4.2)	1.0 [1.0; 2.0]	n/a	2 [1; 3]	3 [2; 8]	36 [20; 45]
**Dimethyl fumarate vs. placebo**								
CONFIRM				Data for the relevant subpopulation were not provided				
DEFINE				Data for the relevant subpopulation were not provided				

Supplementary Table 5 Disease-specific characteristics of the relevant subpopulations at baseline

Comparison<br>Study<br>Study arm	N	EDSS at baseline<br>Mean (SD)	Time since diagnosis of MS [years]<br>Mean (SD)	Nunber of relapses before baseline<br>Median [Q1; Q3] or (Min; Max)		Number of lesions at baseline<br>Median [Q1; Q3] or (Min; Max)		
				Within 1 year	Within 2 years	T1 Gd+	T1 Hypointense	T2
**Fingolimod vs. placebo**								
FREEDOMS								
Fingolimod	34	2.6 (1.0)	7.4 (6.3)	1.0 [1.0; 1.0]	2.0 [1.0; 3.0]	0 [0; 1]	n/a	n/a
Placebo	28	3.1 (1.8)	6.4 (4.6)	1.0 [1.0; 1.0]	2.0 [1.0; 3.0]	0 [0; 2]	n/a	n/a
FREEDOMS II								
Fingolimod	75	2.7 (1.3)	7.0 (6.0)	1.0 [1.0; 2.0]	2.0 [1.0; 3.0]	0 [0; 1]	n/a	n/a
Placebo	69	2.7 (1.3)	8.1 (6.7)	1.0 [1.0; 2.0]	2.0 [1.5; 3.0]	0 [0; 1]	n/a	n/a
**Teriflunomide vs. placebo**								
TEMSO								
Teriflunomide	39	2.7 (1.2)	8.4 (5.9)	1.0 (0; 4)	2.0 (1; 6)	n/a	n/a	n/a
Placebo	32	2.6 (1.3)	7.3 (5.4)	1.0 (0; 3)	2.0 (1; 7)	n/a	n/a	n/a
TOWER								
Teriflunomide	66	2.9 (1.4)	7.4 (6.2)	1.0 (0; 4)	2.0 (1; 6)	n/a	n/a	n/a
Placebo	68	2.8 (1.4)	6.4 (5.1)	1.0 (0; 4)	2.0 (1;7)	n/a	n/a	n/a
**Studies comparing DMTs directly**								
**Ofatumumab vs. teriflunomide**								
ASCLEPIOS I								
Ofatumumab	121	3.2 (1.3)	7.4 (5.5)	1.0 [1.0; 2.0]	1,0 [0,0; 2.0][a]	0 [0; 1]	n/a	n/a
Teriflunomide	122	3.1 (1.3)	7.5 (5.8)	1.0 [1.0; 2.0]	1.0 [0,0; 2.0][a]	0 [0; 1]	n/a	n/a
ASCLEPIOS II								
Ofatumumab	135	3.1 (1.2)	7.4 (6.5)	1.0 [1.0; 2.0]	1.0 [0.0; 2.0][a]	0 [0; 1]	n/a	n/a
Teriflunomide	147	3.1 (1.3)	7.4 (6.3)	1.0 [1.0; 2.0]	1.0 [0.0; 2.0][a]	0 [0; 1]	n/a	n/a

Supplementary Table 5 Disease-specific characteristics of the relevant subpopulations at baseline

Comparison<br>Study<br>Study arm	N	EDSS at baseline<br>Mean (SD)	Time since diagnosis of MS [years]<br>Mean (SD)	Nunber of relapses before baseline<br>Median [Q1; Q3] or (Min; Max)		Number of lesions at baseline<br>Median [Q1; Q3] or (Min; Max)		
				Within 1 year	Within 2 years	T1 Gd+	T1 Hypointense	T2
**Ponesimod vs. teriflunomide**								
OPTIMUM								
Ponesimod	33	2.7 (1.0)	6.3 (4.7)	1.0 [1.0; 1.0]	2.0 [1.0; 3.0]	1 [1; 5]	n/a	n/a<br>(number of patients with lesions [n %]:<br>< 9: 0 (0)<br>≥ 9: 33 (100))
Teriflunomide	45	3.0 (1.2)	9.4 (6.8)	1.0 [1.0; 1.0]	1.0 [1.0; 2.0]	2 [1; 4]	n/a	n/a<br>(number of patients with lesions [n (%)]:<br>< 9: 1 (2)<br>≥ 9: 44 (98))
**Natalizumab**	No relevant studies were identified.							

a. Refers to the time period of 12 to 24 months prior to screening.

EDSS: Expanded Disability Status Scale; Gd+: gadolinium enriching T1-lesion; IFN: interferon; Max: Maximum; Min: Minimum; MS: multiple sclerosis; N: number of randomised patients; n: number of patients with event; n/a: not available; Q1: 1st quartile; Q3: 3rd quartile; SD: standard deviation; vs.: versus

Supplementary Table 6 Results for the outcomes "number of patients with confirmed relapse" (RR, 95% CI), "time to disability progression by EDSS" (HR, 95% CI), and "treatment discontinuation due to AE" (RR, 95% CI) from analyses in patients with highly active RRMS despite previous DMT

**Comparison of DMTs (horizontal vs. vertical)**	**Alemtuzumab**	**Cladribine**	**Dimethyl fumarate**	**Fingolimod**	**Natalizumab**	**Ocrelizumab**	**Ofatumumab**	**Ozanimod**	**Ponesimod**	**Teriflunomide**
**Alemtuzumab**		–	–	–	–	x	–	–	–	–
**Cladribine**	–		x	–	–	–	–	–	–	–
**Dimethyl fumarate**	–	x		x	–	–	x	–	x	x
**Fingolimod**	–	–	x		–	–	Confirmed relapse: 1.08 [0.55; 2.12] Disability progression: 2.09 [0.44; 10.00] Discontinuation due to AE: –	–	–	Confirmed relapse: 0.65 [0.37; 1.17] Disability progression: 0.98 [0.25; 3.88] Discontinuation due to AE: –
**Natalizumab**	–	–	–	–		–	–	–	–	–
**Ocrelizumab**	x	–	–	–	–		–	x	–	–
**Ofatumumab**	–	–	x	Confirmed relapse: 0.92 [0.47; 1.81] Disability progression: 0.48 [0.10; 2.28] Discontinuation due to AE: –	–	–		–	–	**Confirmed relapse: 0.60 [0.43; 0.86] Disability progression: 0.47 [0.22; 0.98] Discontinuation due to AE: 0.32 [0.13; 0.78]**[a]
**Ozanimod**	–	–	–	–	–	x	–		–	–
**Ponesimod**	–	–	x	–	–	–	–	–		Confirmed relapse: 0.61 [0.32; 1.17] Disability progression: 0.17 [0.02; 1.47] **Discontinuation due to AE: 4.77 [1.06; 21.52]**[a]

Supplementary Table 6 Results for the outcomes "number of patients with confirmed relapse" (RR, 95% CI), "time to disability progression by EDSS" (HR, 95% CI), and "treatment discontinuation due to AE" (RR, 95% CI) from analyses in patients with highly active RRMS despite previous DMT

**Comparison of DMTs (horizontal vs. vertical)**	**Alemtuzumab**	**Cladribine**	**Dimethyl fumarate**	**Fingolimod**	**Natalizumab**	**Ocrelizumab**	**Ofatumumab**	**Ozanimod**	**Ponesimod**	**Teriflunomide**
**Teriflunomide**	–	–	x	Confirmed relapse: 1.53 [0.86; 2.72]<br>Disability progression: 1.02 [0.26; 4.06]<br>Discontinuation due to AE: –	–	–	**Confirmed relapse: 1.65 [1.17; 2.34]**<br>**Disability progression: 2.14 [1.02; 4.49]**<br>**Discontinuation due to AE: 3.14 [1.29; 7.64]**[a]	–	Confirmed relapse: 1.63 [0.86; 3.11]<br>Disability progression: 6.02 [0.68; 53.39]<br>**Discontinuation due to AE: 0.21 [0.05; 0.94]**[a]	

Entries printed in **bold** indicate statistically significant effects.
–: Based on the available studies, either no comparative analysis is possible, or results are not interpretable, because only one study with high risk of bias on at least one comparison of an indirect comparison was available.
x: Data for the subpopulation of patients with highly active RRMS despite previous DMT were not provided.
a. Result from direct comparison between the two DMTs.

AE: adverse event; CI: confidence interval; DMT: disease modifying therapy; EDSS: Expanded Disability Status Scale; HR: hazard ratio; RR: relative risk; RRMS: relapsing-remitting multiple sclerosis

Supplementary Table 7 Results for the outcomes MSFC (z-score: MD, 95% CI) and T25FW (metres: MD, 95% CI) from analyses in patients with highly active RRMS despite previous DMT

Comparison of DMTs (horizontal vs. vertical	Alemtuzumab	Cladribine	Dimethyl fumarate	Fingolimod	Natalizumab	Ocrelizumab	Ofatumumab	Ozanimod	Ponesimod	Teriflunomide
**Alemtuzumab**		–	–	–	–	x	–	–	–	–
**Cladribine**	–		x	–	–	–	–	–	–	–
**Dimethyl fumarate**	–	x		x	–	–	x	–	x	x
**Fingolimod**	–	–	x		–	–	x	–	–	x
**Natalizumab**	–	–	–	–		–	–	–	–	–
**Ocrelizumab**	x	–	–	–	–		–	x	–	–
**Ofatumumab**	–	–	x	x	–	–		–	MSFC: -0.13 [-0.29; 0.03]<br>T25FW: 1.03 [-0.22; 2.28]	MSFC: -0.01 [-0.10; 0.07][a]<br>T25FW: 0.57 [-0.35; 1.48][a]
**Ozanimod**	–	–	–	–	–	x	–		–	–
**Ponesimod**	–	–	x	–	–	–	MSFC: 0.13 [-0.03; 0.29]<br>T25FW: -1.03 [-2.28; 0.22]	–		MSFC: 0.12 [-0.02; 0.25][a]<br>T25FW: -0.46 [-1.31; 0.40][a]
**Teriflunomide**	–	–	x	x	–	–	MSFC: 0.01 [-0.07; 0.10][a]<br>T25FW: -0.57 [-1.48; 0.35][a]	–	MSFC: -0.12 [-0.25; 0.02][a]<br>T25FW: 0.46 [-0.40; 1.31][a]	

Entries printed in **bold** indicate statistically significant effects.
–: Based on the available studies, either no comparative analysis is possible, or results are not interpretable, because only one study with high risk of bias on at least one comparison of an indirect comparison was available.
x: Data for the subpopulation of patients with highly active disease RRMS previous DMT were not provided.
a. Result from direct comparison between the two DMTs.

CI: confidence interval; DMT: disease modifying therapy; MD: mean difference from baseline; MSFC: Multiple Sclerosis Functional Composite; RRMS: relapsing-remitting multiple sclerosis; T25FW: Timed 25-foot Walk Test

Supplementary Table 8 Results for the outcome health-related quality of life (SF-36: RR, 95% CI) from analyses in patients with highly active RRMS despite previous DMT

Comparison of DMTs (horizontal vs. vertical)	Alemtuzumab	Cladribine	Dimethyl fumarate	Fingolimod	Natalizumab	Ocrelizumab	Ofatumumab	Ozanimod	Ponesimod	Teriflunomide
**Alemtuzumab**		–	–	–	–	x	–	–	–	–
**Cladribine**	–		x	–	–	–	–	–	–	–
**Dimethyl fumarate**	–	x		x	–	–	x	–	x	x
**Fingolimod**	–	–	x		–	–	–	–	–	–
**Natalizumab**	–	–	–	–		–	–	–	–	–
**Ocrelizumab**	x	–	–	–	–		–	x	–	–
**Ofatumumab**	–	–	x	–	–	–		–	–	–
**Ozanimod**	–	–	–	–	–	x	–		–	–
**Ponesimod**	–	–	x	–	–	–	–	–		MCS (improvement)[a]: 1.58 [0.69; 3.65][c]<br>PCS (improvement)[b]: 1.93 [0.40; 9.19][c]<br>MCS (deterioration)[a]: 0.66 [0.23; 1.94][c]<br>PCS (deterioration)[b]: 0.30 [0.06; 1.41][c]
**Teriflunomide**	–	–	x	–	–	–	–	–	MCS (improvement)[a]: 0.63 [0.27; 1.45][c]<br>PCS (improvement)[b]: 0.52 [0.11; 2.50][c]<br>MCS (deterioration)[a]: 1.52 [0.52; 4.35][c]<br>PCS (deterioration)[b]: 3.33 [0.71; 16.67][c]	

Entries printed in **bold** indicate statistically significant effects.
–: Based on the available studies, either no comparative analysis is possible, or results are not interpretable, because only one study with high risk of bias on at least one comparison of an indirect comparison was available.
x: Data for the subpopulation of patients with highly active RRMS despite previous DMT were not provided.
a. Change by ≥ 15% of the scale range (10.8 points).
b. Change by ≥ 15% of the scale range (10.05 points).
c. Direct comparison.

CI: confidence interval; DMT: disease modifying therapy; MCS: Mental Component Summary; PCS: Physical Component Summary; RR: relative risk; RRMS: relapsing-remitting multiple sclerosis; SF-36: Short Form 36

Supplementary Table 9 Studies without results published up to July 2023

Study	Study ID [Reference]	Planned sample size	Study status[a] (Study Completion, if available)
**Cladribin**			
ChariotMS	NCT04695080 [269], 2018-005038-39 [270]	200	ongoing (12/2027)
NOR-MS	NCT04121403 [271], 2019-001505-24 [272]	264	ongoing (12/2024)
**Ocrelizumab**			
BN42082	NCT04544436 [273], 2020-000893-69 [274], PER-056-20 [275]	865	ongoing (08/2028)
DanNORMS	NCT04688788 [276], 2020-002981-15 [277]	594	ongoing (04/2028)
**Teriflunomide**			
BCD-132-2	NCT04056897 [278]	270	unclear[a]
evolutionRMS 1 (MS200527_0080)	NCT04338022 [279], 2019-004972-20 [280], CTRI/2020/10/028457 [281]	1124	ongoing (06/2026)
evolutionRMS 2 (MS200527_0082)	NCT04338061 [282], 2019-004980-36 [283], CTRI/2020/10/028183 [284]	1124	ongoing (06/2026)
FENhance (GN41851)	NCT04586010 [285], 2019-004857-10 [286], PER-076-20 [287]	736	ongoing (11/2025)
FENhance 2 (GN42272)	NCT04586023 [288], 2020-001168-28 [289], CTRI/2021/03/031904 [290]	736	ongoing (11/2025)
GEMINI 1	NCT04410978 [291], 2020-000637-41 [292]	900	ongoing (09/2023)
GEMINI 2	NCT04410991 [293], 2020-000644-55 [294], CTRI/2020/11/029237 [295]	900	ongoing (08/2023)
a. status available in the registry on 22th July 2023<br>b. registries were checked in July 2023, last update in 2021, study was labelled ongoing			

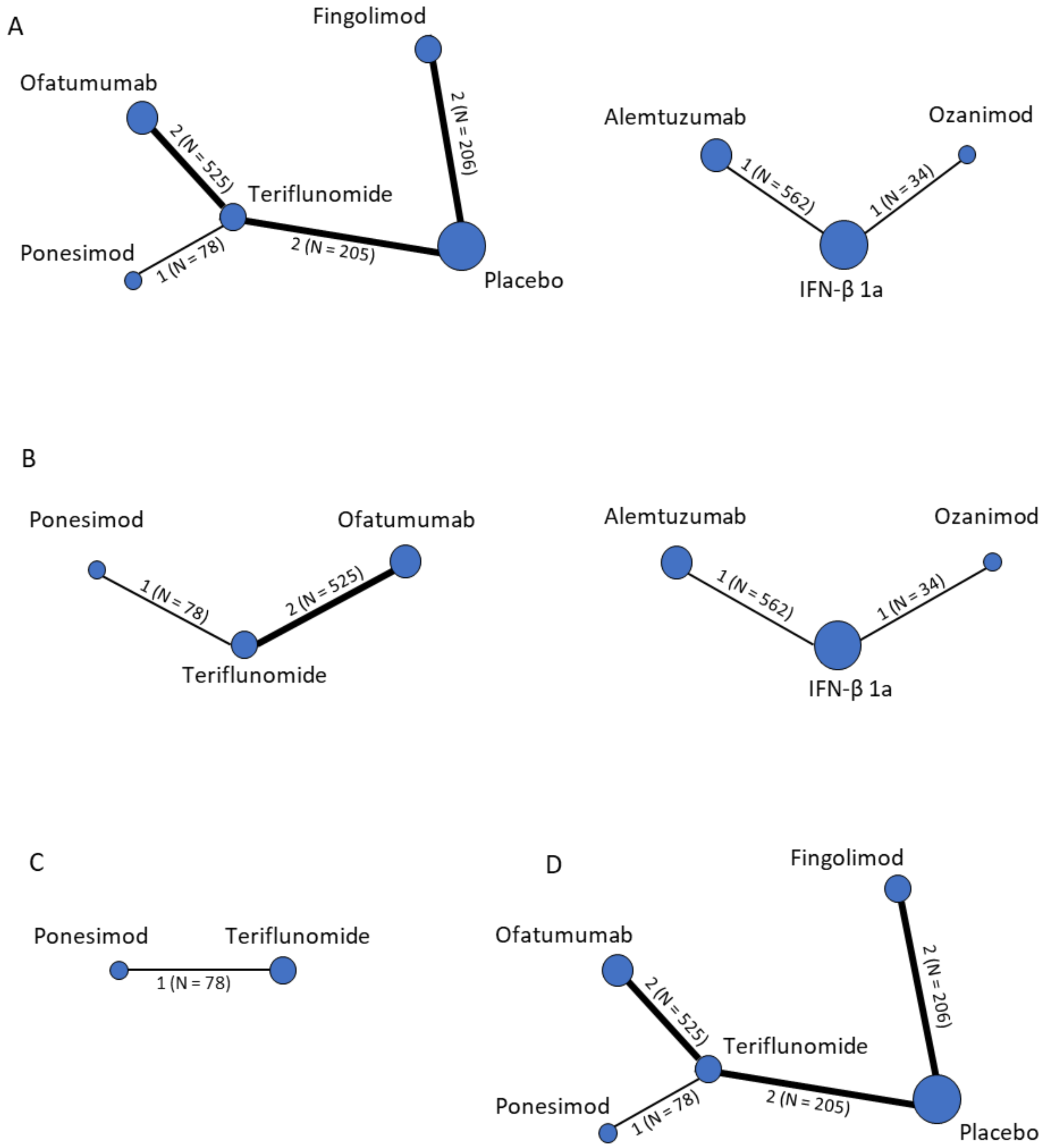

Supplementary Figure 1 Comparisons for outcomes other than relapse rate and serious adverse events for which data were available.

A: EDSS-based disability progression, confirmed after 24 weeks, B: severity of disability (assessed with MSFC z-score) and walking ability (assessed with T25FW), C: health-related quality of life, D: discontinuation due to adverse events. Numbers indicate the number of studies per comparison and total number of patients. EDSS = Expanded Disability Status Scale; IFN = interferon; MSFC = Multiple Sclerosis Functional Composite; N = total number of patients; T25FW = Timed 25-Foot Walk Test

Literature

1. Coles AJ, Twyman CL, Arnold DL (2012) Alemtuzumab for patients with relapsing multiple sclerosis after disease-modifying therapy: a randomised controlled phase 3 trial. Lancet 380(9856):1829-1839. https://doi.org/10.1016/s0140-6736(12)61768-1.

2. Arnold DL, Fisher E, Brinar VV (2016) Superior MRI outcomes with alemtuzumab compared with subcutaneous interferon beta-1a in MS. Neurology 87(14):1464-1472. https://doi.org/10.1212/wnl.0000000000003169.

3. Arroyo Gonzalez R, Kita M, Crayton H (2017) Alemtuzumab improves quality-of-life outcomes compared with subcutaneous interferon beta-1a in patients with active relapsing-remitting multiple sclerosis. Multiple Sclerosis 23(10):1367-1376. https://doi.org/10.1177/1352458516677589.

4. Giovannoni G, Cohen JA, Coles AJ (2016) Alemtuzumab improves preexisting disability in active relapsing-remitting MS patients. Neurology 87(19):1985-1992. https://doi.org/10.1212/wnl.0000000000003319.

5. Ziemssen T, Bass AD, Berkovich R (2020) Efficacy and Safety of Alemtuzumab Through 9 Years of Follow-up in Patients with Highly Active Disease: Post Hoc Analysis of CARE-MS I and II Patients in the TOPAZ Extension Study. CNS Drugs 34(9):973-988. https://doi.org/10.1007/s40263-020-00749-x.

6. Genzyme a Sanofi Company (2017) Comparison of Alemtuzumab and Rebif Efficacy in Multiple Sclerosis, Study Two (CARE-MS II). https://ClinicalTrials.gov/show/NCT00548405. Accessed 12.05.2021.

7. Genzyme A Phase 3 Randomized, Rater- and Dose-Blinded Study Comparing Two Annual Cycles of Intravenous Low- and High-Dose Alemtuzumab to Three-Times Weekly Subcutaneous Interferon Beta-1a (Rebif) in Patients with Relapsing-Remitting Multiple Scleroris Who Have Relapsed On Therapy. https://www.clinicaltrialsregister.eu/ctr-search/search?query=eudract_number:2007-001162-32. Accessed 18.05.2021.

8. Hauser SL, Bar-Or A, Comi G (2017) Ocrelizumab versus Interferon Beta-1a in Relapsing Multiple Sclerosis. New England Journal of Medicine 376(3):221-234. https://doi.org/10.1056/NEJMoa1601277.

9. Barkhof F, Kappos L, Wolinsky JS (2019) Onset of clinical and MRI efficacy of ocrelizumab in relapsing multiple sclerosis. Neurology 93(19):e1778-e1786. https://doi.org/10.1212/wnl.0000000000008189.

10. Mayer L, Kappos L, Racke MK (2019) Ocrelizumab infusion experience in patients with relapsing and primary progressive multiple sclerosis: Results from the phase 3 randomized OPERA I, OPERA II, and ORATORIO studies. Multiple Sclerosis and Related Disorders 30:236-243. https://doi.org/10.1016/j.msard.2019.01.044.

11. Turner B, Cree BAC, Kappos L (2019) Ocrelizumab efficacy in subgroups of patients with relapsing multiple sclerosis. Journal of Neurology 266(5):1182-1193. https://doi.org/10.1007/s00415-019-09248-6.

12. Wolinsky JS, Engmann NJ, Pei J (2020) An exploratory analysis of the efficacy of ocrelizumab in patients with multiple sclerosis with increased disability. Mult Scler J Exp Transl Clin 6(1):2055217320911939. https://doi.org/10.1177/2055217320911939.

13. Kappos L, Wolinsky JS, Giovannoni G (2020) Contribution of Relapse-Independent Progression vs Relapse-Associated Worsening to Overall Confirmed Disability Accumulation in Typical Relapsing Multiple Sclerosis in a Pooled Analysis of 2 Randomized Clinical Trials. JAMA Neurol 77(9):1132-1140. https://doi.org/10.1001/jamaneurol.2020.1568.

14. Hoffmann-La Roche (2020) A Study of Ocrelizumab in Comparison With Interferon Beta-1a (Rebif) in Participants With Relapsing Multiple Sclerosis. https://ClinicalTrials.gov/show/NCT01247324. Accessed 14.05.2021.

15. F. Hoffmann-La Roche A Randomized, Double-Blind, Double-Dummy, Parallel-Group Study To Evaluate The Efficacy And Safety Of Ocrelizumab In Comparison To Interferon Beta-1a (Rebif®) In Patients With Relapsing Multiple Sclerosis. https://www.clinicaltrialsregister.eu/ctr-search/search?query=eudract_number:2010-020337-99. Accessed 18.05.2021.

16. F. Hoffmann La Roche / Genentech A Randomized, Double-Blind, Double-Dummy, Parallel-Group Study to Evaluate the Efficacy and Safety of Ocrelizumab in Comparison to Interferon Beta-1a (Rebif®) in Patients with Relapsing Multiple Sclerosis. https://www.ins.gob.pe/ensayosclinicos/rpec/recuperarECPBNuevoEN.asp?numec=024-14. Accessed 18.05.2021.

17. F. Hoffmann La Roche / Genentech A Randomized, Double-Blind, Double-Dummy, Parallel-Group Study to Evaluate the Efficacy and Safety of Ocrelizumab in Comparison to Interferon Beta-1a (REBIF) in Patients with Relapsing Multiple Sclerosis. https://www.ins.gob.pe/ensayosclinicos/rpec/recuperarECPBNuevoEN.asp?numec=128-11. Accessed 18.05.2021.

18. MacMillan EL, Schubert JJ, Vavasour IM (2019) Magnetic resonance spectroscopy evidence for declining gliosis in MS patients treated with ocrelizumab versus interferon beta-1a. Mult Scler J Exp Transl Clin 5(4):2055217319879952. https://doi.org/10.1177/2055217319879952.

19. Hoffmann-La Roche (2020) A Study of Ocrelizumab in Comparison With Interferon Beta-1a (Rebif) in Participants With Relapsing Multiple Sclerosis. https://ClinicalTrials.gov/show/NCT01412333. Accessed 14.06.2021.

20. F. Hoffmann-La Roche A Randomized, Double-Blind, Double-Dummy, Parallel-Group Study To Evaluate The Efficacy And Safety Of Ocrelizumab In Comparison To Interferon Beta-1a (REBIF) In Patients With Relapsing Multiple Sclerosis. https://www.clinicaltrialsregister.eu/ctr-search/search?query=eudract_number:2010-020315-36. Accessed 18.05.2021.

21. Cohen JA, Comi G, Selmaj KW (2019) Safety and efficacy of ozanimod versus interferon beta-1a in relapsing multiple sclerosis (RADIANCE): a multicentre, randomised, 24-month, phase 3 trial. Lancet Neurology 18(11):1021-1033. https://doi.org/10.1016/s1474-4422(19)30238-8.

22. Harris S, Comi G, Cree BAC (2021) Plasma neurofilament light chain concentrations as a biomarker of clinical and radiologic outcomes in relapsing multiple sclerosis: Post hoc analysis of Phase 3 ozanimod trials. European Journal of Neurology 28(11):3722-3730. https://doi.org/10.1111/ene.15009.

23. Celgene (2021) Efficacy and Safety Study of Ozanimod in Relapsing Multiple Sclerosis (RADIANCE). https://ClinicalTrials.gov/show/NCT02047734. Accessed 15.02.2022.

24. Receptos A Phase 2/3, Multi-Center, Randomized, Double-Blind, Placebo Controlled (Part a) and Double-Blind, Double-Dummy, Active Controlled (Part B), Parallel Group Study to Evaluate the Efficacy and Safety of RPC1063 Administered Orally to Relapsing Multiple Sclerosis Patients. https://www.clinicaltrialsregister.eu/ctr-search/search?query=eudract_number:2012-002714-40. Accessed 15.02.2022.

25. Giovannoni G, Comi G, Cook S (2010) A placebo-controlled trial of oral cladribine for relapsing multiple sclerosis. New England Journal of Medicine 362(5):416-426. https://doi.org/10.1056/NEJMoa0902533.

26. Afolabi D, Albor C, Zalewski L (2018) Positive impact of cladribine on quality of life in people with relapsing multiple sclerosis. Multiple Sclerosis 24(11):1461-1468. https://doi.org/10.1177/1352458517726380.

27. Ali S, Paracha N, Cook S (2012) Reduction in healthcare and societal resource utilization associated with cladribine tablets in patients with relapsing-remitting multiple sclerosis: analysis of economic data from the CLARITY Study. Clinical Drug Investigation 32(1):15-27. https://doi.org/10.2165/11593310-000000000-00000.

28. Comi G, Cook SD, Giovannoni G (2013) MRI outcomes with cladribine tablets for multiple sclerosis in the CLARITY study. Journal of Neurology 260(4):1136-1146. https://doi.org/10.1007/s00415-012-6775-0.

29. Cook S, Vermersch P, Comi G (2011) Safety and tolerability of cladribine tablets in multiple sclerosis: the CLARITY (CLAdRIbine Tablets treating multiple sclerosis orallY) study. Multiple Sclerosis 17(5):578-593. https://doi.org/10.1177/1352458510391344.

30. De Stefano N, Giorgio A, Battaglini M (2018) Reduced brain atrophy rates are associated with lower risk of disability progression in patients with relapsing multiple sclerosis treated with cladribine tablets. Multiple Sclerosis 24(2):222-226. https://doi.org/10.1177/1352458517690269.

31. Giovannoni G, Cook S, Rammohan K (2011) Sustained disease-activity-free status in patients with relapsing-remitting multiple sclerosis treated with cladribine tablets in the CLARITY study: a post-hoc and subgroup analysis. Lancet Neurology 10(4):329-337. https://doi.org/10.1016/s1474-4422(11)70023-0.

32. Giovannoni G, Soelberg Sorensen P, Cook S (2019) Efficacy of Cladribine Tablets in high disease activity subgroups of patients with relapsing multiple sclerosis: A post hoc analysis of the CLARITY study. Multiple Sclerosis 25(6):819-827. https://doi.org/10.1177/1352458518771875.

33. Hermann R, Litwin JS, Friberg LE (2019) Effects of cladribine tablets on heart rate, atrio-ventricular conduction and cardiac repolarization in patients with relapsing multiple sclerosis. British Journal of Clinical Pharmacology 85(7):1484-1494. https://doi.org/10.1111/bcp.13919.

34. Rammohan K, Giovannoni G, Comi G (2012) Cladribine tablets for relapsing-remitting multiple sclerosis: Efficacy across patient subgroups from the phase III CLARITY study. Multiple Sclerosis and Related Disorders 1(1):49-54. https://doi.org/10.1016/j.msard.2011.08.006.

35. Terranova N, Hicking C, Dangond F, Munafo A (2019) Effects of Postponing Treatment in the Second Year of Cladribine Administration: Clinical Trial Simulation Analysis of Absolute Lymphocyte Counts and Relapse Rate in Patients with Relapsing-Remitting Multiple Sclerosis. Clinical Pharmacokinetics 58(3):325-333. https://doi.org/10.1007/s40262-018-0693-y.

36. Vermersch P, Galazka A, Dangond F (2020) Efficacy of cladribine tablets in high disease activity patients with relapsing multiple sclerosis: Post hoc analysis of subgroups with and without prior disease-modifying drug treatment. Current Medical Research & Opinion:1. https://doi.org/10.1080/03007995.2020.1865888.

37. De Stefano N, Sormani MP, Giovannoni G (2021) Analysis of frequency and severity of relapses in multiple sclerosis patients treated with cladribine tablets or placebo: The CLARITY and CLARITY Extension studies. Multiple Sclerosis:13524585211010294. https://doi.org/10.1177/13524585211010294.

38. Giovannoni G, Comi G, Rammohan K (2021) Long-Term Disease Stability Assessed by the Expanded Disability Status Scale in Patients Treated with Cladribine Tablets 3.5 mg/kg for Relapsing Multiple Sclerosis: An Exploratory Post Hoc Analysis of the CLARITY and CLARITY Extension Studies. Advances in Therapy 38(9):4975-4985. https://doi.org/10.1007/s12325-021-01865-w.

39. EMD Serono (2014) A Safety and Efficacy Study of Oral Cladribine in Subjects With Relapsing-remitting Multiple Sclerosis (RRMS) (CLARITY). https://ClinicalTrials.gov/show/NCT00213135. Accessed 14.05.2021.

40. Merck Serono International A phase III, randomised, double-blind, three-arm, placebo-controlled, multi-center study to evaluate the safety and efficacy of oral Cladribine in subjects with relapsing remitting multiple sclerosis. https://www.clinicaltrialsregister.eu/ctr-search/search?query=eudract_number:2004-005148-28. Accessed 14.05.2021.

41. Fox RJ, Miller DH, Phillips JT (2012) Placebo-controlled phase 3 study of oral BG-12 or glatiramer in multiple sclerosis. New England Journal of Medicine 367(12):1087-1097. https://doi.org/10.1056/NEJMoa1206328.

42. Freedman MS, Montalban X, Miller AE (2016) Comparing outcomes from clinical studies of oral disease-modifying therapies (dimethyl fumarate, fingolimod, and teriflunomide) in relapsing MS: Assessing absolute differences using a number needed to treat analysis. Multiple Sclerosis and Related Disorders 10:204-212. https://doi.org/10.1016/j.msard.2016.10.010.

43. Havrdova E, Giovannoni G, Gold R (2017) Effect of delayed-release dimethyl fumarate on no evidence of disease activity in relapsing-remitting multiple sclerosis: integrated analysis of the phase III DEFINE and CONFIRM studies. European Journal of Neurology 24(5):726-733. https://doi.org/10.1111/ene.13272.

44. Hutchinson M, Fox RJ, Miller DH (2013) Clinical efficacy of BG-12 (dimethyl fumarate) in patients with relapsing-remitting multiple sclerosis: subgroup analyses of the CONFIRM study. Journal of Neurology 260(9):2286-2296. https://doi.org/10.1007/s00415-013-6968-1.

45. Kita M, Fox RJ, Phillips JT (2014) Effects of BG-12 (dimethyl fumarate) on health-related quality of life in patients with relapsing-remitting multiple sclerosis: findings from the CONFIRM study. Multiple Sclerosis 20(2):253-257. https://doi.org/10.1177/1352458513507818.

46. Miller DH, Fox RJ, Phillips JT (2015) Effects of delayed-release dimethyl fumarate on MRI measures in the phase 3 CONFIRM study. Neurology 84(11):1145-1152. https://doi.org/10.1212/wnl.0000000000001360.

47. Biogen (2015) Efficacy and Safety Study of Oral BG00012 With Active Reference in Relapsing-Remitting Multiple Sclerosis (CONFIRM). https://ClinicalTrials.gov/show/NCT00451451. Accessed 14.05.2021.

48. Biogen Idec A Randomized, Multicenter, Placebo-Controlled and Active Reference (Glatiramer Acetate) Comparison Study to Evaluate the Efficacy and Safety of BG00012 in Subjects With Relapsing-Remitting Multiple Sclerosis. https://www.clinicaltrialsregister.eu/ctr-search/search?query=eudract_number:2006-003697-10. Accessed 14.05.2021.

49. Biogen Idec (2013) A Randomized, Multicenter, Placebo-Controlled and Active Reference (Glatiramer Acetate) Comparison Study to Evaluate the Efficacy and Safety of BG00012 in Subjects With Relapsing-Remitting Multiple Sclerosis. http://www.ctri.nic.in/Clinicaltrials/pmaindet2.php?trialid=380. Accessed 18.05.2021.

50. Gold R, Kappos L, Arnold DL (2012) Placebo-controlled phase 3 study of oral BG-12 for relapsing multiple sclerosis. New England Journal of Medicine 367(12):1098-1107. https://doi.org/10.1056/NEJMoa1114287.

51. Arnold DL, Gold R, Kappos L (2014) Effects of delayed-release dimethyl fumarate on MRI measures in the Phase 3 DEFINE study. Journal of Neurology 261(9):1794-1802. https://doi.org/10.1007/s00415-014-7412-x.

52. Arnold DL, Gold R, Kappos L (2014) Magnetization transfer ratio in the delayed-release dimethyl fumarate DEFINE study. Journal of Neurology 261(12):2429-2437. https://doi.org/10.1007/s00415-014-7504-7.

53. Bar-Or A, Gold R, Kappos L (2013) Clinical efficacy of BG-12 (dimethyl fumarate) in patients with relapsing-remitting multiple sclerosis: subgroup analyses of the DEFINE study. Journal of Neurology 260(9):2297-2305. https://doi.org/10.1007/s00415-013-6954-7.

54. Kappos L, Gold R, Arnold DL (2014) Quality of life outcomes with BG-12 (dimethyl fumarate) in patients with relapsing-remitting multiple sclerosis: the DEFINE study. Multiple Sclerosis 20(2):243-252. https://doi.org/10.1177/1352458513507817.

55. Biogen (2015) Efficacy and Safety of Oral BG00012 in Relapsing-Remitting Multiple Sclerosis (DEFINE). https://ClinicalTrials.gov/show/NCT00420212. Accessed 14.05.2021.

56. Biogen Idec A Randomized, Multicenter, Double-Blind, Placebo-Controlled, Dose-Comparison Study to Determine the Efficacy and Safety of BG00012 in Subjects with Relapsing-Remitting Multiple Sclerosis. https://www.clinicaltrialsregister.eu/ctr-search/search?query=eudract_number:2006-003696-12. Accessed 14.05.2021.

57. Biogen Idec (2015) A Randomized, Multicenter, Double-Blind, Placebo-Controlled, Dose-Comparison Study to Determine the Efficacy and Safety of BG00012 in Subjects With Relapsing-Remitting Multiple Sclerosis. http://www.ctri.nic.in/Clinicaltrials/pmaindet2.php?trialid=356. Accessed 18.05.2021.

58. Biogen Idec (2007) A Randomized, Multicentre, Double-Blind, Placebo-Controlled, Dose-Comparison Study to Determine the Safety and Efficacy of BG00012 in Subjects with Relapsing Remitting Multiple Sclerosis. https://anzctr.org.au/ACTRN12607000124437.aspx. Accessed 18.05.2021.

59. Kappos L, Radue EW, O'Connor P (2010) A placebo-controlled trial of oral fingolimod in relapsing multiple sclerosis. New England Journal of Medicine 362(5):387-401. https://doi.org/10.1056/NEJMoa0909494.

60. Agius M, Meng X, Chin P (2014) Fingolimod therapy in early multiple sclerosis: an efficacy analysis of the TRANSFORMS and FREEDOMS studies by time since first symptom. CNS Neuroscience & Therapeutics 20(5):446-451. https://doi.org/10.1111/cns.12235.

61. Devonshire V, Havrdova E, Radue EW (2012) Relapse and disability outcomes in patients with multiple sclerosis treated with fingolimod: subgroup analyses of the double-blind, randomised, placebo-controlled FREEDOMS study. Lancet Neurology 11(5):420-428. https://doi.org/10.1016/s1474-4422(12)70056-x.

62. Gartner J, Chitnis T, Ghezzi A (2018) Relapse Rate and MRI Activity in Young Adult Patients With Multiple Sclerosis: A Post Hoc Analysis of Phase 3 Fingolimod Trials. Mult Scler J Exp Transl Clin 4(2):2055217318778610. https://doi.org/10.1177/2055217318778610.

63. Jeffery DR, Di Cantogno EV, Ritter S (2016) The relationship between the rate of brain volume loss during first 24 months and disability progression over 24 and 48 months in relapsing MS. Journal of Neurology 263(2):299-305. https://doi.org/10.1007/s00415-015-7959-1.

64. Kremenchutzky M, O'Connor P, Hohlfeld R (2014) Impact of prior treatment status and reasons for discontinuation on the efficacy and safety of fingolimod: Subgroup analyses of the Fingolimod Research Evaluating Effects of Daily Oral Therapy in Multiple Sclerosis (FREEDOMS) study. Multiple Sclerosis and Related Disorders 3(3):341-349. https://doi.org/10.1016/j.msard.2013.10.006.

65. Kuhle J, Disanto G, Lorscheider J (2015) Fingolimod and CSF neurofilament light chain levels in relapsing-remitting multiple sclerosis. Neurology 84(16):1639-1643. https://doi.org/10.1212/wnl.0000000000001491.

66. Kuhle J, Kropshofer H, Haering DA (2019) Blood neurofilament light chain as a biomarker of MS disease activity and treatment response. Neurology 92(10):e1007-e1015. https://doi.org/10.1212/wnl.0000000000007032.

67. Radue EW, Barkhof F, Kappos L (2015) Correlation between brain volume loss and clinical and MRI outcomes in multiple sclerosis. Neurology 84(8):784-793. https://doi.org/10.1212/wnl.0000000000001281.

68. Radue EW, O'Connor P, Polman CH (2012) Impact of fingolimod therapy on magnetic resonance imaging outcomes in patients with multiple sclerosis. Archives of Neurology 69(10):1259-1269. https://doi.org/10.1001/archneurol.2012.1051.

69. Sormani MP, De Stefano N, Francis G (2015) Fingolimod effect on brain volume loss independently contributes to its effect on disability. Multiple Sclerosis 21(7):916-924. https://doi.org/10.1177/1352458515569099.

70. Sormani MP, Haering DA, Kropshofer H (2019) Blood neurofilament light as a potential endpoint in Phase 2 studies in MS. Ann Clin Transl Neurol 6(6):1081-1089. https://doi.org/10.1002/acn3.795.

71. Vermersch P, Radue EW, Putzki N (2017) A comparison of multiple sclerosis disease activity after discontinuation of fingolimod and placebo. Mult Scler J Exp Transl Clin 3(3):2055217317730096. https://doi.org/10.1177/2055217317730096.

72. Novartis (2012) Efficacy and Safety of Fingolimod in Patients With Relapsing-remitting Multiple Sclerosis (FREEDOMS). https://ClinicalTrials.gov/show/NCT00289978. Accessed 14.05.2021.

73. Novartis Pharma Services A 24-month, double-blind, randomized, multicenter, placebo-controlled, parallel-group study comparing efficacy and safety of FTY720 1.25 mg and 0.5 mg administered orally once daily versus placebo in patients with relapsing-remitting multiple sclerosis. https://www.clinicaltrialsregister.eu/ctr-search/search?query=eudract_number:2005-000365-19. Accessed 14.05.2021.

74. Calabresi PA, Radue EW, Goodin D (2014) Safety and efficacy of fingolimod in patients with relapsing-remitting multiple sclerosis (FREEDOMS II): a double-blind, randomised, placebo-controlled, phase 3 trial. Lancet Neurology 13(6):545-556. https://doi.org/10.1016/s1474-4422(14)70049-3.

75. Winges KM, Werner JS, Harvey DJ (2013) Baseline retinal nerve fiber layer thickness and macular volume quantified by OCT in the North American phase 3 fingolimod trial for relapsing-remitting multiple sclerosis. Journal of Neuro-Ophthalmology 33(4):322-329. https://doi.org/10.1097/WNO.0b013e31829c51f7.

76. Novartis (2012) Efficacy and Safety of Fingolimod (FTY720) in Patients With Relapsing-remitting Multiple Sclerosis (FREEDOMS II). https://ClinicalTrials.gov/show/NCT00355134. Accessed 14.05.2021.

77. Novartis Pharma Services A 24-month double-blind, randomized, multicenter, placebocontrolled, parallel-group study comparing the efficacy and safety of 0.5 mg and 1.25 mg fingolimod (FTY720) administered orally once daily versus placebo in patients with relapsing-remitting multiple sclerosis. https://www.clinicaltrialsregister.eu/ctr-search/search?query=eudract_number:2008-002096-27. Accessed 14.05.2021.

78. O'Connor P, Wolinsky JS, Confavreux C (2011) Randomized trial of oral teriflunomide for relapsing multiple sclerosis. New England Journal of Medicine 365(14):1293-1303. https://doi.org/10.1056/NEJMoa1014656.

79. Miller AE, O'Connor P, Wolinsky JS (2012) Pre-specified subgroup analyses of a placebo-controlled phase III trial (TEMSO) of oral teriflunomide in relapsing multiple sclerosis. Multiple Sclerosis 18(11):1625-1632. https://doi.org/10.1177/1352458512450354.

80. O'Connor PW, Lublin FD, Wolinsky JS (2013) Teriflunomide reduces relapse-related neurological sequelae, hospitalizations and steroid use. Journal of Neurology 260(10):2472-2480. https://doi.org/10.1007/s00415-013-6979-y.

81. Wolinsky JS, Narayana PA, Nelson F (2013) Magnetic resonance imaging outcomes from a phase III trial of teriflunomide. Multiple Sclerosis 19(10):1310-1319. https://doi.org/10.1177/1352458513475723.

82. Sprenger T, Kappos L, Radue EW (2020) Association of brain volume loss and long-term disability outcomes in patients with multiple sclerosis treated with teriflunomide. Multiple Sclerosis 26(10):1207-1216. https://doi.org/10.1177/1352458519855722.

83. Sanofi (2013) Study of Teriflunomide in Reducing the Frequency of Relapses and Accumulation of Disability in Patients With Multiple Sclerosis (TEMSO). https://ClinicalTrials.gov/show/NCT00134563. Accessed 14.05.2021.

84. Sanofi-Aventis US A randomized, double-blind, placebo-controlled, parallel group design study to evaluate the efficacy and safety of teriflunomide (HMR1726D) in reducing the frequency of relapses and delaying the accumulation of physical disability in subjects with multiple sclerosis with relapses. https://www.clinicaltrialsregister.eu/ctr-search/search?query=eudract_number:2004-000555-42. Accessed 14.05.2021.

85. Confavreux C, O'Connor P, Comi G (2014) Oral teriflunomide for patients with relapsing multiple sclerosis (TOWER): a randomised, double-blind, placebo-controlled, phase 3 trial. Lancet Neurology 13(3):247-256. https://doi.org/10.1016/s1474-4422(13)70308-9.

86. Miller AE, Macdonell R, Comi G (2014) Teriflunomide reduces relapses with sequelae and relapses leading to hospitalizations: results from the TOWER study. Journal of Neurology 261(9):1781-1788. https://doi.org/10.1007/s00415-014-7395-7.

87. Miller AE, Xu X, Macdonell R (2019) Efficacy and safety of teriflunomide in Asian patients with relapsing forms of multiple sclerosis: A subgroup analysis of the phase 3 TOWER study. Journal of Clinical Neuroscience 59:229-231. https://doi.org/10.1016/j.jocn.2018.09.012.

88. Qiu W, Huang DH, Hou SF (2018) Efficacy and Safety of Teriflunomide in Chinese Patients with Relapsing Forms of Multiple Sclerosis: A Subgroup Analysis of the Phase 3 TOWER Study. Chinese Medical Journal 131(23):2776-2784. https://doi.org/10.4103/0366-6999.246067.

89. Sanofi (2016) An Efficacy Study of Teriflunomide in Participants With Relapsing Multiple Sclerosis (TOWER). https://ClinicalTrials.gov/show/NCT00751881. Accessed 14.05.2021.

90. Sanofi-Aventis Recherche & Développement A multi-center double-blind parallel-group placebo-controlled study of the efficacy and safety of teriflunomide in patients with relapsing multiple sclerosis. https://www.clinicaltrialsregister.eu/ctr-search/search?query=eudract_number:2007-004452-36. Accessed 14.05.2021.

91. Sanofi-Aventis Recherche & Development A multi-center double-blind parallel-group placebo-controlled study of the efficacy and safety of teriflunomide in patients with relapsing multiple sclerosis. https://www.ins.gob.pe/ensayosclinicos/rpec/recuperarECPBNuevoEN.asp?numec=111-08. Accessed 15.02.2022.

92. Hauser SL, Bar-Or A, Cohen JA (2020) Ofatumumab versus Teriflunomide in Multiple Sclerosis. New England Journal of Medicine 383(6):546-557. https://doi.org/10.1056/NEJMoa1917246.

93. Novartis Pharmaceuticals (2021) Efficacy and Safety of Ofatumumab Compared to Teriflunomide in Patients With Relapsing Multiple Sclerosis (ASCLEPIOS I). https://ClinicalTrials.gov/show/NCT02792218. Accessed 15.02.2022.

94. Novartis Healthcare (2019) A randomized, double-blind, double-dummy, parallel-group study comparing the efficacy and safety of ofatumumab versus teriflunomide in patients with relapsing multiple sclerosis. http://www.ctri.nic.in/Clinicaltrials/pmaindet2.php?trialid=16236. Accessed 15.02.2022.

95. Novartis Pharma A randomized, double-blind, double-dummy, parallel-group study comparing the efficacy and safety of ofatumumab versus teriflunomide in patients with relapsing multiple sclerosis. https://www.clinicaltrialsregister.eu/ctr-search/search?query=eudract_number:2015-005418-31. Accessed 11.02.2022.

96. Novartis Pharmaceuticals (2021) Efficacy and Safety of Ofatumumab Compared to Teriflunomide in Patients With Relapsing Multiple Sclerosis. (ASCLEPIOS II). https://ClinicalTrials.gov/show/NCT02792231. Accessed 15.02.2022.

97. Novartis Biosciences Peru A randomized, double-blind, double-dummy, parallel-group study comparing the efficacy and safety of ofatumumab versus teriflunomide in patients with relapsing multiple sclerosis. https://www.ins.gob.pe/ensayosclinicos/rpec/recuperarECPBNuevoEN.asp?numec=049-16. Accessed 11.02.2022.

98. Novartis Pharma Services A randomized, double-blind, double-dummy, parallel-group study comparing the efficacy and safety of ofatumumab versus teriflunomide in patients with relapsing multiple sclerosis. https://www.clinicaltrialsregister.eu/ctr-search/search?query=eudract_number:2015-005419-33. Accessed 11.02.2022.

99. Novartis Healthcare (2019) A randomized, double-blind, double-dummy, parallel-group study comparing the efficacy and safety of ofatumumab versus teriflunomide in patients with relapsing multiple sclerosis. http://www.ctri.nic.in/Clinicaltrials/pmaindet2.php?trialid=16414. Accessed 15.02.2022.

100. Kappos L, Fox RJ, Burcklen M (2021) Ponesimod Compared With Teriflunomide in Patients With Relapsing Multiple Sclerosis in the Active-Comparator Phase 3 OPTIMUM Study: A Randomized Clinical Trial. JAMA Neurology 78(5):558-567. https://doi.org/10.1001/jamaneurol.2021.0405.

101. Actelion (2021) Oral Ponesimod Versus Teriflunomide In Relapsing MUltiple Sclerosis (OPTIMUM). https://ClinicalTrials.gov/show/NCT02425644. Accessed 11.02.2022.

102. Actelion Pharmaceuticals Multicenter, randomized, double-blind, parallel-group, active-controlled, superiority study to compare the efficacy and safety of ponesimod to teriflunomide in subjects with relapsing multiple sclerosis. https://www.clinicaltrialsregister.eu/ctr-search/search?query=eudract_number:2012-000540-10. Accessed 15.02.2022.

103. Salanti G, Higgins JP, Ades AE, Ioannidis JP (2008) Evaluation of networks of randomized trials. Stat Methods Med Res 17(3):279-301. https://doi.org/10.1177/0962280207080643.

104. Donegan S, Williamson P, D'Alessandro U, Tudur Smith C (2013) Assessing key assumptions of network meta-analysis: a review of methods. Res Synth Methods 4(4):291-323. https://doi.org/10.1002/jrsm.1085.

105. Kiefer C, Sturtz S, Bender R (2020) A simulation study to compare different estimation approaches for network meta-analysis and corresponding methods to evaluate the consistency assumption. BMC Med Res Methodol 20(1):36. https://doi.org/10.1186/s12874-020-0917-3.

106. Salanti G (2012) Indirect and mixed-treatment comparison, network, or multiple-treatments meta-analysis: many names, many benefits, many concerns for the next generation evidence synthesis tool. Res Synth Methods 3(2):80-97. https://doi.org/10.1002/jrsm.1037.

107. Hutton B, Salanti G, Caldwell DM (2015) The PRISMA extension statement for reporting of systematic reviews incorporating network meta-analyses of health care interventions: checklist and explanations. Ann Intern Med 162(11):777-784. https://doi.org/10.7326/M14-2385.

108. Efthimiou O, Debray TP, van Valkenhoef G (2016) GetReal in network meta-analysis: a review of the methodology. Res Synth Methods 7(3):236-263. https://doi.org/10.1002/jrsm.1195.

109. Cope S, Zhang J, Saletan S (2014) A process for assessing the feasibility of a network meta-analysis: a case study of everolimus in combination with hormonal therapy versus chemotherapy for advanced breast cancer. BMC Med 12:93. https://doi.org/10.1186/1741-7015-12-93.

110. Deeks JJ HJ, Altman DG (2023) Analysing data and undertaking meta-analyses. https://training.cochrane.org/handbook/current/chapter-10. Accessed 28 February 2024.

111. Janke K, Biester K, Krause D (2020) Comparative effectiveness of biological medicines in rheumatoid arthritis: systematic review and network meta-analysis including aggregate results from reanalysed individual patient data. BMJ 370:m2288. https://doi.org/10.1136/bmj.m2288.

112. Rücker G (2012) Network meta-analysis, electrical networks and graph theory. Res Synth Methods 3(4):312-324. https://doi.org/10.1002/jrsm.1058.

113. Balduzzi S, Rücker G, Nikolakopoulou A (2023) netmeta: An R Package for Network Meta-Analysis Using Frequentist Methods. J Stat Softw 106(2):1-40. https://doi.org/10.18637/jss.v106.i02.

114. Veroniki AA, Straus SE, Rucker G, Tricco AC (2018) Is providing uncertainty intervals in treatment ranking helpful in a network meta-analysis? J Clin Epidemiol 100:122-129. https://doi.org/10.1016/j.jclinepi.2018.02.009.

115. Salanti G, Nikolakopoulou A, Mavridis D (2019) On ranking mulitiple health interventions. https://www.dagstat2019.statistik.uni-muenchen.de/funktionen/pdfs/book_of_abstracts2019.pdf. Accessed 17 April 2024.

116. Mills EJ, Thorlund K, Ioannidis JP (2013) Demystifying trial networks and network meta-analysis. BMJ 346:f2914. https://doi.org/10.1136/bmj.f2914.

117. Mbuagbaw L, Rochwerg B, Jaeschke R (2017) Approaches to interpreting and choosing the best treatments in network meta-analyses. Syst Rev 6(1):79. https://doi.org/10.1186/s13643-017-0473-z.

118. Institut für Qualität und Wirtschaftlichkeit im Gesundheitswesen (2023) Alemtuzumab, Cladribin, Dimethylfumarat, Fingolimod, Natalizumab, Ocrelizumab, Ofatumumab, Ozanimod, Ponesimod und Teriflunomid zur Behandlung Erwachsener mit hochaktiver schubförmig remittierender multipler Sklerose; Abschlussbericht. https://www.iqwig.de/download/a20-60_multiple-sklerose_abschlussbericht_v1-0.pdf. Accessed 10 October 2023.
